# Current View on Phytoplasma Genomes and Encoded Metabolism

**DOI:** 10.1100/2012/185942

**Published:** 2011-12-05

**Authors:** Michael Kube, Jelena Mitrovic, Bojan Duduk, Ralf Rabus, Erich Seemüller

**Affiliations:** ^1^Department of Crop and Animal Sciences, Humboldt-University of Berlin, Lentzeallee 55/57, 14195 Berlin, Germany; ^2^Max Planck Institute for Molecular Genetics, Ihnestr. 63, 14195 Berlin, Germany; ^3^Department of Plant Pathology, Institute of Pesticides and Environmental Protection, Banatska 31b, P.O. Box 163, 11080 Belgrade, Serbia; ^4^Institute for Chemistry and Biology of the Marine Environment, Carl von Ossietzky University of Oldenburg, Carl-von-Ossietzky Straße 9-11, 26111 Oldenburg, Germany; ^5^Department for Microbiology, MaxPlanck Institute for Marine Microbiology, Celsiusstraße 1, 28359 Bremen, Germany; ^6^Institute for Plant Protection in Fruit Crops and Viticulture, Federal Research Centre for Cultivated Plants, Schwabenheimer Straße 101, 69221 Dossenheim, Germany

## Abstract

Phytoplasmas are specialised bacteria that are obligate parasites of plant phloem tissue and insects. These bacteria have resisted all attempts of cell-free cultivation. Genome research is of particular importance to analyse the genetic endowment of such bacteria. Here we review the gene content of the four completely sequenced ‘*Candidatus* Phytoplasma' genomes that include those of ‘*Ca.* P. asteris' strains OY-M and AY-WB, ‘*Ca.* P. australiense,' and ‘*Ca.* P. mali'. These genomes are characterized by chromosome condensation resulting in sizes below 900 kb and a G + C content of less than 28%. Evolutionary adaption of the phytoplasmas to nutrient-rich environments resulted in losses of genetic modules and increased host dependency highlighted by the transport systems and limited metabolic repertoire. On the other hand, duplication and integration events enlarged the chromosomes and contribute to genome instability. Present differences in the content of membrane and secreted proteins reflect the host adaptation in the phytoplasma strains. General differences are obvious between different phylogenetic subgroups. ‘*Ca.* P. mali' is separated from the other strains by its deviating chromosome organization, the genetic repertoire for recombination and excision repair of nucleotides or the loss of the complete energy-yielding part of the glycolysis. Apart from these differences, comparative analysis exemplified that all four phytoplasmas are likely to encode an alternative pathway to generate pyruvate and ATP.

## 1. Introduction

**Figure 1:**
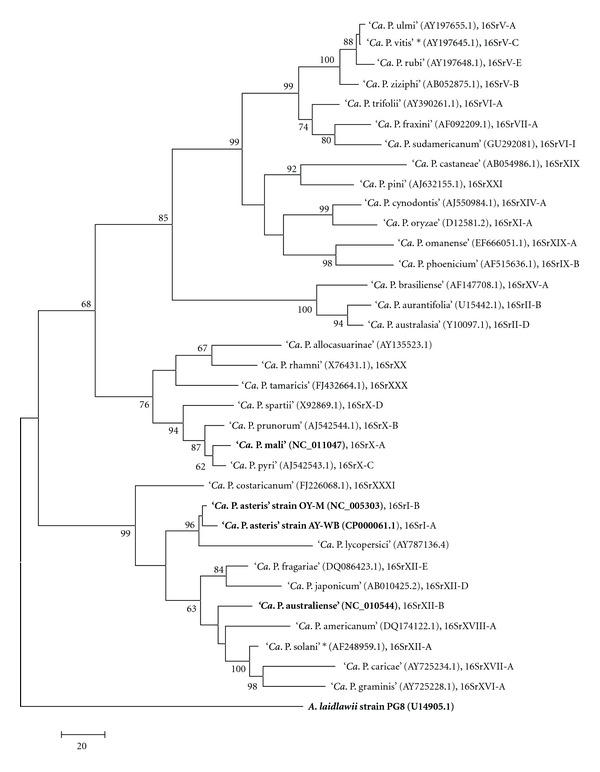
Phylogenetic tree constructed by parsimony analyses of 16S rDNA sequences of 34 phytoplasma strains, belonging to all ‘*Candidatus* Phytoplasma' species described employing *A. laidlawii* as outgroup. The completely sequenced strains are in bold. CLUSTAL W from the Molecular Evolutionary Genetics Analysis program-MEGA5 [26] was used for multiple alignment. The maximum parsimony tree was obtained using the Close-Neighbour-Interchange algorithm, implemented in the MEGA5, with search level 3 in which the initial trees were obtained with the random addition of sequences (10 replicates). One of nine equally parsimonious trees is shown in Figure 1. Numbers on the branches are bootstrap values obtained for 2000 replicates (only values above 60% are shown). GenBank accession numbers are given in parentheses, and 16Sr group classification (when available) is shown on the right side of the tree. Asterisk indicates ‘*Candidatus* Phytoplasma' proposed by the IRPCM Phytoplasma/Spiroplasma Working Team—phytoplasma taxonomy group [5], not yet formally described. The tree is drawn to scale with branch lengths calculated using the average pathway method and represents the number of changes over the whole sequence. The scale bar represents 20 nucleotide substitutions.

Phytoplasmas are bacteria which colonise plants and some groups of insects. They are associated with diseases of several hundred plant species including important crops [1]. Just for apple, economical losses caused by ‘*Candidatus* Phytoplasma mali' and related phytoplasmas of 100 million €/year in Italy and Germany were estimated [2]. Phytoplasmas are pleomorphic but nonhelical, 0.2–0.8 *μ*m in diameter, and lack, as members of the class *Mollicutes*, a firm cell wall. Phytoplasmas were first called mycoplasma-like organisms on discovery in 1967 [3]. Today, they are grouped in the provisional genus ‘*Candidatus* Phytoplasma' that is assigned to a family of *incertae sedis *[4]. Thirty-two ‘*Candidatus* Phytoplasma' species have been described [5–7]. The phytoplasma clade is divided into two major subclades and numerous subgroups based on the 16S rRNA marker gene (Figure 1). Based on their 16S rDNA sequence they are phylogenetically assigned to a distinct monophyletic taxon in the *Mollicutes* as member of the order* Acholeplasmatales *[8]. *Mollicutes* are deeply branching from the clostridial lineage leading to the genera *Bacillus* and *Lactobacillus *[9–11]. They are assigned to the phylum *Tenericutes*. Within the mollicutes, phytoplasmas are most closely related to the genus *Acholeplasma* [12, 13] with which they share, in contrast to other mollicutes, the usage of UGA as a stop codon [14]. Based on 16S rDNA analysis, Weisburg et al. [15] proposed that acholeplasmas were formed early at the initial divergence of the *Mollicutes* from clostridial ancestors, followed by genome condensation and loss of the cell wall. Another divergence may have occurred that led to the separation of the phytoplasmas from the acholeplasmas. Genome condensation took place in both groups resulting in genomes sizes of about 1.215–2.095 kb and 27–38 mol% GC for the acholeplasmas [16, 17] and 530–1.350 kb and 21–33 mol% GC for the phytoplasmas [4, 17–20].

Information on the phytoplasma host environment is essential for understanding their adaptation and genome condensation. In plants, phytoplasmas mainly colonise the phloem sieve elements, but have occasionally also been reported to occur in companion and parenchyma cells as well [21–23]. The sieve tube environment is highly appropriate for the pathogens because it offers excellent conditions for the spread in the plant host. The phytoplasmas can readily pass the sieve pores of the sieve tubes and may be passively translocated according to the source/sink principle of the phloem sap flow in the various growing seasons [1, 24, 25].

The phloem fulfills functions in long-distance transport and allocation of nutrients and emits signals related to nutrition, development, and stress response. Probably the phloem transport functions by mass flow through the sieve elements and by cell-to-cell transport by surrounding companion cells and phloem parenchyma cells that provide metabolites to the sap [27].

Phloem sap is unique for several reasons. It is under high hydrostatic pressure and rich in nutrients. The content varies depending on the plant species but always contains large amounts of carbohydrates. The most abundant one is often sucrose (12–30%) beside other sugars such as glucose and fructose, minerals, proteins, amino acids, and ATP [28, 29]. Other sugars such as polyols and oligosaccharides of the raffinose family may be also abundant, depending on the species [30]. Sugar alcohols such as mannitol and sorbitol may also be present in considerable amount [31, 32].

The osmotic pressure of the sieve tube sap ranges from 0.8–2.5 MP and the pH is usually between 7.3 and 8.5 [30]. Bicarbonate and malate may play a role in the control of phloem sap pH [33, 34]. It is notable that malate and citrate represent the predominant organic acids in phloem and xylem sap [35], and one may speculate whether the content of these organic acids is regulated as a consequence of phytoplasma infection. It is known that the pH value of media used for cultivation of plant-derived isolates of *Acholeplasma brassicae* and *A. palmae* decreases significantly during growth.

Other nutrients such as nitrate, inorganic phosphate, glutathione, glutamate, and amino acids were also detected in phloem sap [36, 37]. For lettuce (*Lactuca sativa* L.) it was shown [28] that fourteen amino acids are present (Asp, Thr, Ser, Asn, Glu, Gln, Gly, Ala, Val, Ile, Leu, Tyr, Phe, and gamma-isobutyric acid/GABA). Glutamine, glutamic acid, serine, and GABA were often predominant. The concentration in lettuce was 125 mM for all amino acids. The presence of high ammonia concentrations (6 mM) is also remarkable. However, depending on the method also arginine and trace quantities of alpha-aminobutyric-acids, methionine, tryptophan, and ornithine were detected. Secondary substances such as phenolics, flavonoids, sesquiterpene lactones, alkaloids, and sterols have also been reported [28].

Some of the listed compounds and others such as cytokinins, auxins, abscisic acid, gibberellins, jasmonates, and methyl salicylates [27, 36, 37] and small RNAs [38, 39] highlight the importance of the phloem for signalling. It is still under discussion if the phloem is also a location for protein synthesis [27]. The determination of pumpkin sap proteome supports this idea [40].

Phytoplasmas are mainly transmitted by phloem-feeding insects such as leafhoppers, planthoppers, and psyllids [41, 42]. Under certain conditions, vectors may be attracted by plants releasing plant allomones manipulated by the phytoplasma infection [42]. They have to acquire the pathogen by phloem feeding [43, 44]. Subsequently, the phytoplasmas multiply in the hemolymph in which the concentration of organic compounds is similarly high than in phloem sap [45]. It is remarkable that trehalose is the predominant sugar in the hemolymph of aphids while glucose appeared as an artefact, and sucrose was not detected [45]. The trehalose content of the hemolymph of the phloem-sucking insects is correlated to the sucrose content of the aphid's host plants [45]. Interaction of phytoplasmas with their insect vectors has been reported for ‘*Ca*. P. asteris' strains. Dominant membrane proteins such as the major antigenic protein Amp interact with the vector proteins such as actin and the alpha and beta subunits of the ATP synthase [46]. The insect environment in combination with the ability to migrate from the insect gut to the salivary glands allows the spread of the pathogen [47].

Four complete phytoplasma genomes have been published. However, there is no recent publication summarizing the encoded repertoire of these genomes (see also ST, supplementary table in Supplementary Material available online at doi: 10.1100/2012/185942) and discussing the deduced metabolism. For this reason this paper attempts to fill this gap by reviewing published results and re-evaluating the genomic data.

## 2. Genomic Benchmarks

The first fully sequenced genome was that of the onion yellows phytoplasma strain OY-M of subgroup 16SrI-B [48]. It was followed by the closely related aster yellows witches'-broom phytoplasma strain AY-WB of subgroup 16SrI-A that showed large regions of conserved synteny [49]. In addition to the information on this two ‘*Ca*. P. asteris' genomes the sequences of two members from other phylogenetic groups were published in 2008, namely, that of ‘*Ca*. P. australiense' [50] and that of ‘*Ca*. P. mali' [20] (Table 1). With a size of 880 kb ‘*Ca*. P. australiense' shows the largest phytoplasma chromosome deciphered so far while ‘*Ca*. P. mali' with a size of 602 kb stands at the opposite end belonging to the second major subclade. ‘*Ca*. P. mali' is characterized by the extremely low G + C content of less than 22%, while the other three genomes show a similar G + C content of 27-28%. The number of protein-encoding genes ranges corresponding to their genome sizes from 481 to 776 but may be also influenced by different annotation styles. The highest number of strain-specific genes is present in ‘*Ca*. P. australiense' [50]. The only available genome sequence from the related genus *Acholeplasma* is that of *A. laidlawii* strain PG-8A (Acc. no. CP000896), which differs from phytoplasma genomes by a larger size (1.5 Mb), higher number of protein-encoding genes (1,380), and a higher G + C content (32%). However, all sequenced phytoplasmas belong to three subgroups leaving most subgroups out of this analyses (Figure 1).

The reason for the low number of completely sequenced phytoplasma genomes is not surprising if one considers the difficulties encountered. Mainly due to the problem in obtaining suitable DNA for sequencing, it took years to complete ‘*Ca*. P. asteris' strain OY-M and ‘*Ca*. P. mali' strain AT sequencing. Because phytoplasma DNA usually has to be extracted from infected plants in which it occurs at very low concentration, elaborate enrichment and purification procedures were required to obtain it in amounts of DNA necessary for a high coverage of each position of the genome in shotgun sequencing. Flowers of *Catharanthus roseus* (periwinkle) and phloem of greenhouse-grown *Nicotiana tabacum* (tobacco) plants were preferentially used to obtain DNA templates for ‘*Ca*. P. australiense' and ‘*Ca*. P. mali' sequencing, respectively. The amount of phytoplasma DNA obtained depends on the enrichment procedure used. For example, when ‘*Ca*. P. mali' DNA was, after extraction from *N. occidentalis*, treated by repeated bisbenzimide-CsCl buoyant density gradient centrifugation, a 30% enrichment of phytoplasma DNA was obtained. In contrast, purifying of phloem extract from *N. tabacum* using pulsed-field gel electrophoresis resulted in 80% enrichment [20]. Starting from an enriched phytoplasma DNA template, the genome sequences were determined by a shotgun approach [51]. In theory, shotgun fragments and the derived sequences represent the whole genome equally. However, AT-rich regions show a decreased physical coverage by recombinant clones/shotgun sequences and a lower sequence quality. Low coverage regions in the genomes are the result. This problem also arises if new strategies from next generation technologies such as pyrosequencing [52] are used. The effect is reduced by these clone-independent approaches and high sequence coverage.

The assembly and finishing of the repeat-rich genomes represents an additional problem. It remains time consuming because clone-based large-insert libraries of phytoplasmas (e.g. fosmid libraries) do show a high cloning bias and are thus limited in their suitability for finishing experiments. The generation of additional complete genome sequence will remain a challenge in near future, but, hopefully, decreasing sequencing costs, high sequencing coverage, mate-pair libraries, and enlarged read lengths averaging 800 bases derived from pyrosequencing will help to overcome these problems.

 The four phytoplasma genomes are organized in chromosomes and often contain extrachromosomal elements, which are reported for several phytoplasma strains [53]. The extrachromosomal elements, often called plasmids, allow the integration of their genetic material into the chromosome [49] and remarkably influence the vector transmissibility as shown for some strains [54]. Transmission experiments also indicate that plasmid-like extrachromosomal elements may not belong to the stable content of the genome. 

The chromosomes of nearly all *Mollicutes* including those of the two ‘*Ca*. P. asteris' strains [48, 49], ‘*Ca*. P. australiense' [50], and *A. laidlawii *are circular. In contrast, ‘*Ca*. P. mali' and the closely related species ‘*Ca*. P. pyri' (pear decline phytoplasma) and ‘*Ca*. P. prunorum' (European stone fruit yellows phytoplasmas) have a linear chromosome [20]. It may indicate a characteristic of this phylogenetic cluster. The chromosome of ‘*Ca*. P. mali' has terminal inverted repeats (TIRs) with a size of 43 kb that have covalently closed hairpin ends protecting the chromosome [20]. TIRs are also known from *Streptomyces* genomes and covalently closed hairpin ends occur in genera *Borellia *and *Coxiella*, and several linear plasmids and phages [55]. A candidate gene encoding a required telomere resolvase (ResT) has been suggested to occur in ‘*Ca*. P. mali' chromosome (ATP_00103) [20]. However, further experiments are needed to clarify the proposed function. Within the TIR region neither tRNAs nor rRNAs genes are encoded. The origin of this unusual chromosome organisation within the *Mollicutes* and its influence on the replication process in phytoplasmas remains unclear. One may speculate whether these structures have a virus-related origin. 

Viruses influence the phytoplasma genomes. Wei and colleagues proposed that phages of the order *Caudovirales* have a major impact on the two fully sequenced ‘*Ca*. P. asteris' genomes [56]. They analysed the deviations in the conserved synteny of these two closely related genomes and the encoded cryptic phage-derived genes and calculated that 264.2 kb (~31% of the genome) of strain OY-M and 160.2 kb (~22.7% of the genome) of strain AY-WB are putatively of viral origin. The Prophage Finder was one of the central bioinformatical tools used [56, 57]. Employing the Prophage Finder approach to examine the four fully sequenced phytoplasma genomes results in similar percentages of phage-derived sequences and shows 32% for strain OY-M, 20% for strain AY-WB, 26% for ‘*Ca*. P. australiense' and 26% for ‘*Ca*. P. mali'. Even if such an approach can just give a raw estimation, it is obvious that these genomes are heavily influenced by integration events. Genome diversity of phytoplasmas was also shown in other studies, for example, by PFGE analysis and mapping of marker genes of the sweet potato little leaf (SPLL) disease strain V4 (SPLL-V4) and of the closely related tomato big bud (TBB) phytoplasma [58]. 

Genome instability of phytoplasmas is not restricted to phage integration and recombination events or exchangeable plasmid material. Complex transposons, called potential mobile units (PMUs) [49], have significant impact in duplication of parts of phytoplasma genomes. PMUs are suggested to act as a tool to generate genetic variability [49]. Recently, it was shown that they might form extrachromosomal elements that may replicate and integrate again into phytoplasma genomes [59]. A high portion of the encoded proteins in the four genomes was assigned to these elements. Hogenhout and Musić [60] assigned 486 (OY-M) to 408 (‘*Ca*. P. mali') proteins of the four phytoplasma genomes as single copy genes. This means that 35% of all protein-encoding genes in OY-M are multicopy genes. The corresponding proteins encoded by multicopy genes were assigned to PMUs. This indicates that 65–70% of them of ‘*Ca*. P. asteris' strains AY-WB and OY-M and of ‘*Ca*. P. australiense' but only 4% of ‘*Ca*. P. mali' genes are encoded in such structures. Even if this provisional assignment of genes to phages or PMUs will show overlaps, there is no doubt that both events took place in phytoplasma genomes and that it seems unlikely that PMUs are the major driving force for duplicated genes in ‘*Ca*. P. mali' that encodes only one *tra5* transposase as central element of PMUs. Interestingly, no PMUs have been identified in *A. laidlawii*. 

**Figure 2:**
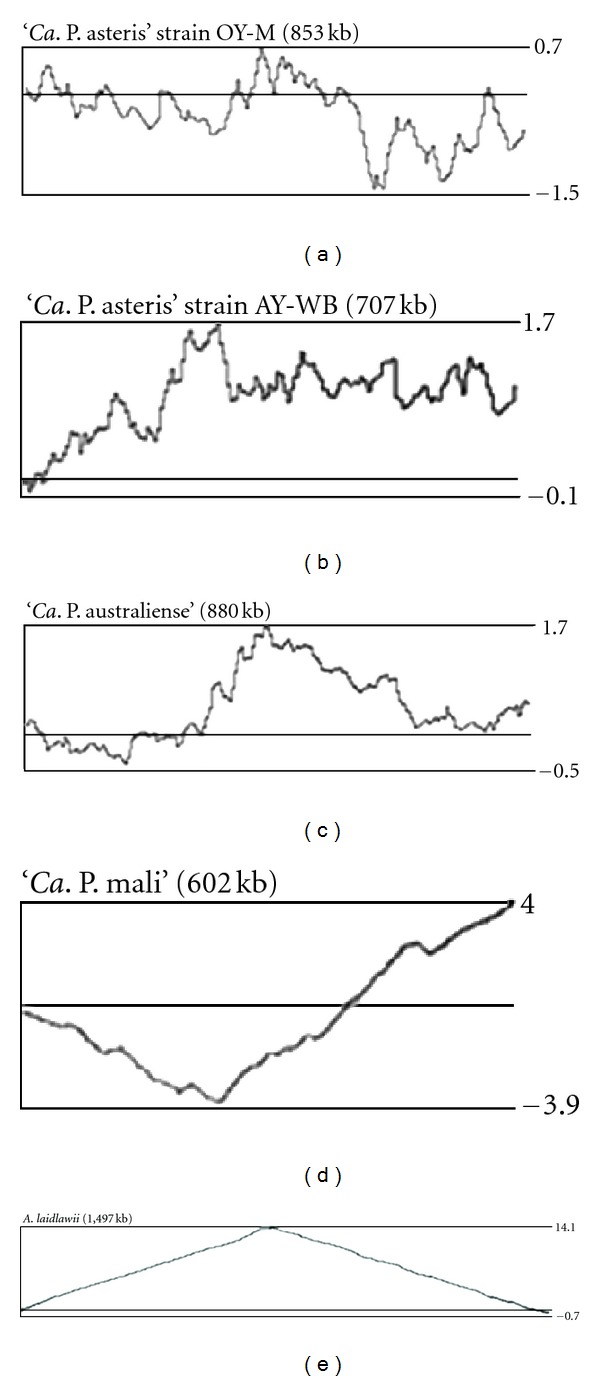
Cumulative GC skew analysis [(C − G)/(G + C)] of the chromosomes of the two ‘*Ca*. P. asteris' strains OY-M and AY-WB, ‘*Ca*. P. australiense', ‘*Ca*. P. mali,' and *A. laidlawii*. A window size of 5000 bases was used for calculation. Maxima and minima values obtained are indicated.

The impact of other genetic elements such as reverse transcriptases remains unclear. At least ‘*Ca*. P. asteris' strain OY-M and ‘*Ca*. P. australiense' encode a reverse transcriptase (ST1). The presence of such a gene usually indicates a mobile element such as a retrotransposon or retrovirus [61]. The result of these integration and recombination events can be visualized by cumulative GC analysis that shows irregular skews for the ‘*Ca*. P. asteris' strains and ‘*Ca*. P. australiense' but only in parts for ‘*Ca*. P. mali' and not for *A. laidlawii *(Figure 2). 

All these events enlarge the condensed phytoplasma genomes [49], but the enlarged genome size does not necessarily expand the number of encoded functions. 

**Figure 3:**
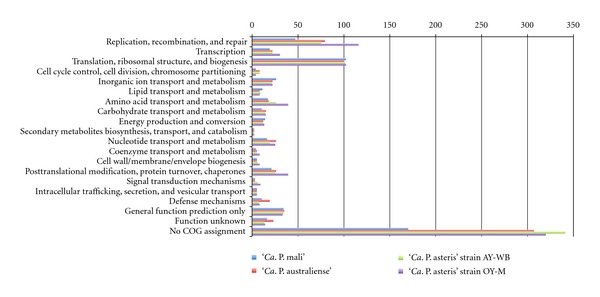
COG assignment of the deduced proteins of the four phytoplasma genomes. Single deduced proteins can show more than one assignment to the COGs database.

Different levels of genome condensation [20] and the putative phage-derived horizontal transfers of genetic material [56] or transposon-driven duplication events [49] result in different genome sizes in phytoplasmas. Insights for the COG (cluster of orthologous groups) assignment [62] of the protein-coding content of the four phytoplasmas show (Figure 3) that the majority of the functional categories is affected by these driving mechanisms and gives the impression that they share only for few categories a similar content. The largest category without any COG assignment may indicate the individual evolution of each phytoplasma but also an amount of genes, which are resulting from overprediction or by representing pseudogenes. The analysis of the shared genetic content provides insights into the general demands on the host while differences may reflect strain-specific capabilities. 

## 3. Replication, Recombination, and Repair

### 3.1. Replication Proteins and *oriC*

All essential genes necessary to generate the bacterial replication fork are encoded in the four genomes. These compromise the proteins forming the prereplisome such as DnaA, which binds at the *oriC*, the interacting DnaB/DnaC complex, and DnaD [63]. The phytoplasmas also share the DNA gyrase (GyrAB) and the DnaB helicase performing the bidirectional DNA unwinding [64]. The putative modulator PmbA of the DNA gyrase can influence this process. 

Other encoded proteins, such as DnaG, probably synthesise initial primers at the site of the prereplisome [65], which is needed for the polymerase III heteromultimer. The genes encoding the proteins forming the DNA polymerase III holoenzyme are also present. This protein set contains PolC (alpha subunit, leading strand), DnaE (alpha subunit, lagging strand), DnaN (beta subunit), DnaX (tau subunit), and HolAB (delta and delta prime subunit). The putative assignment of PolC to the leading strand and the DnaE subunit to the lagging strand appears to be likely with respect to the Gram-positive ancestors [15, 66]. 

All phytoplasma genomes encode a NAD-dependent DNA ligase (Lig) for sealing breaks in DNA that occur, for example, during the discontinuous synthesis of the lagging strand. An ATP-dependent DNA ligase has not been annotated, a feature shared with *A. laidlawii *(Acc. no. CP000896). Other proteins involved in replication such as PriA responsible for the restart of stalled replication forks or acting at the occurring RNA/DNA hybrids (RnhC, NrdFA) are also present. 

Some of the genes including *dnaB*, *dnaG*, and others involved in replication such as the single-stranded binding protein (*ssb*), the dna primase (*dnaG*), and the delta subunit of the DNA polymerase III (*polC*) are present as multicopy genes. The origin of these multiple copies is still in discussion but they can be assigned to PMU or phage origin (see Section 2). In spite of this discussion it is notable that the *dnaB* gene is annotated at least twice in phytoplasmas. A situation that is also present in *A. laidlawii* (Acc. no. CP000896). The annotated DnaB2 shows significant differences to DnaB1 in phytoplasmas. DnaB1 contains the DnaB-like helicase N-terminal domain (Pfam entry: PF00772.15) and the corresponding C-terminal domain (Pfam entry: PF03796.9) while *dnaB2* just partially matches the DnaB 2 family entry for replication initiation and membrane attachment (Pfam entry: PF07261.5). A tandem arrangement of *dnaB2* and *dnaC* is encoded in all four genomes. Other tandem arrangements of single copy genes such as *dnaAN *or *gyrAB* and *nrdA/nrdF* are also present in phytoplasmas. 

The coding for the suggested telomere resolvase is restricted to ‘*Ca*. P. mali' [20]. Apart from that *orf*, the genetic environment of the genes involved in replication varies only in the copy number (ST2). 

The *oriC* of the majority of bacterial chromosomes lies close to the overall minimum of the cumulative GC-skew [67, 68]. As phytoplasma chromosomes are heavily influenced by rearrangements and integration events that result in irregular cumulative skews and thus do not show a distinct minimum, an exception is the linear chromosome of ‘*Ca*. P. mali' (Figure 2) [20, 49] that seems to be less influenced by such events and shows a shifted cumulative GC-skew with a minimum close to the sequence upstream the *dnaA* gene (Figure 2) [20]. The other three chromosomes show little pronounced minima close to the location of *dnaA*, indicating the presence of a putative *oriC *[20, 49]. This assignment is supported by several other approaches such as Oriloc [20, 49, 69] or OligoSkew analyses [20] and the typically decreased G + C content at the proposed *oriC*, which is present at the intergenic regions of the phytoplasmas flanking *dnaA*. They contain long AT-stretches of at least 30 bases (31, 33, and 48 bases in strain OY-M; 32 and 48 bases in strain AY-WB; 161 and 39 bases present upstream PAa_0839 in ‘*Ca*. P. australiense'; 30, 33, 43, 87, and 53 bases in ‘*Ca*. P. mali'). AT-stretches were limited in ‘*Ca*. P. australiense' and ‘*Ca*. P. mali' to positions upstream *dnaA* due to the coding density. 

**Figure 4:**
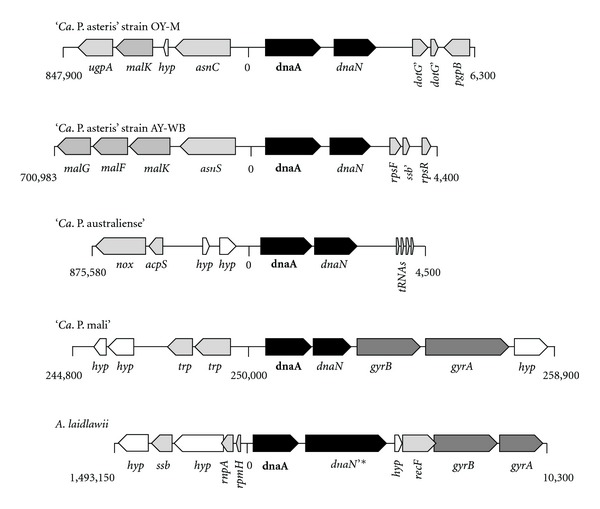
Genomic context at the suggested *oriC*. The *oriC* region is shown for the four phytoplasma chromosomes and *A. laidlawii* illustrating the weak conserved synteny within this region. Several genes are truncated (“ ‘ ”) and one gene probably destroyed by transposase integration (“*”). Abbreviation of genes: hyp: hypothetical protein; trp: ABC transporter subunit. Genome positions were given for each region taking in account the circular organisation of four chromosomes.

The *oriC* regions show only a weakly conserved synteny (Figure 4), a situation similar to that of other *Mollicutes *[70]. A typical switch in the coding strand preference at the *oriC *is present within this region and corresponds to a bidirectional replication. However, ‘*Ca*. P. mali' shows a coding strand preference all over the chromosome [20]. 

The DnaA protein of *Escherichia coli* initially binds a variant of the DnaA-box consensus 5′-TTAT(C/A)CA(C/A)A-3′ at the *oriC* region [71]. DnaA-box motifs flank the *dnaA* gene in several *Mollicutes* [70]. The consensus of the DnaA-box motif of *Mollicutes* such as *M. pulmonis*, *Spiroplasma citri*, *M. capricolum*, and *M. genitalium* (5′-TT(A/T)TC(C/A)ACA-3′) differs from the consensus of *E. coli *in the positions of the variable nucleotides (Table 2) [70]. The seven annotated DnaA-boxes of *A. laidlawii* (Acc. no. CP000896) result in a consensus motif (5′-TT(A/T)T(C/T)(C/A)ACA-3′), which largely matches the suggested motif for other mollicutes [70], but also shows that the fifth position is more variable (A/C/T). Such a consensus sequence of the DnaA-box motif was not identified at the *oriC*-region of the phytoplasma chromosomes. The suggested DnaA-box motif for *Mollicutes *occurs in the neighbourhood of *dnaA* of ‘*Ca*. P. asteris' strain OY-M (position 4878–4886, within the disrupted *dotG* gene; complement 848′134–848′142, encoding also *ugpA* at this position) but not within the intergenic regions flanking the *dnaA* gene. It is absent within the flanking intergenic regions of *dnaA* in strain AY-WB and also in ‘*Ca*. P. australiense', where the occurrence is limited to a region (position 1503–1511) encoding *dnaN*. A similar situation can be found in ‘*Ca*. P. mali'. The mollicute motif is present within the *dnaN* gene (position 252′493–252′501) and within the sequence encoding a putative ABC transporter subunit for methionine (position complement 248′550–248′558). Strain OY-M and ‘*Ca*. P. australiense' share the motif 5′-TTTTCAACA-3′ while ‘*Ca*. P. mali' contains 5′-TTATCAACA-3′. However, it was not possible to identify the proposed mollicute motif in strain AY-WB, its occurrence within sequences encoding genes, and the rare occurrence within this region which raises the question if the consensus (5′-TT(T/A)TCAACA-3′) covers the sequence of the DnaA-box motif. It seems to be likely that the DnaA-box motif shows a higher variability. Other motifs occur with higher frequency such as 5′-ATAGGAACA-3′ (eight times), more interesting 5′-TTAGGAACA-3′ (three times) for ‘*Ca*. P. mali,' and are located within the intergenic regions flanking *dnaA*. The latter is a promising candidate present in all four genomes within the *oriC* region with at least one copy (strain AY-WB) but contains an unusual GG-coding at positions four and five. It is present in the intergenic region of OY-M for four times, of AY-WB once, ‘*Ca*. P. australiense' five times, and ‘*Ca*. P. mali' three times. However, experimental evidence is needed to verify this.

### 3.2. Proteins Involved in Chromosome Partitioning

Segregation and partitioning of the chromosomal DNA start immediately after the initiation of the replication. However, the identified repertoire for this process is sparse, and it is likely that some proteins assigned as conserved hypothetical proteins will be involved. Topoisomerases will decatenate the two chromosome copies. 

All four genomes also encode a regulator belonging to stage V sporulation protein family G (ST3). It is essential for sporulation and specific to stage V sporulation in *Bacillus megaterium* and *B. subtilis *[72]. However, the function in phytoplasma is unknown, and the protein may be involved in other processes. 

In OY-M *smc*-genes were annotated that encode chromosome segregation of ATPase-like proteins. This assignment remains unclear, and the assignment to a glycoprotein appears to be more likely (see below).

### 3.3. Recombination

The complement of genes necessary for resolving Holliday junction and recombination differs in phytoplasmas. ‘*Ca*. P mali' shows the most complete set for *rec*-dependent repair system. It encodes *ruvAB* but the endonuclease *ruvC* was not identified. Instead, RecU may have this function. The RuvABC enzymatic complex binds to recombinational junctions catalysing strand cleavage and branch migration. This is needed for the repair of double-strand breaks, which result from the RecA-dependent pathway. A basic system encoded in ‘*Ca*. P. mali' but not in the other three phytoplasmas consists of recombinase A, the ATP-dependent zinc-containing RecG-like helicase, and the recombinational DNA repair proteins O and R in contrast to the other three phytoplasmas. However, genes *recA*, *recU*, *ruvA*, and *ruvB*, which are absent in three phytoplasmas, do not belong to the essential gene set of *M. genitalium* but may have long-term influence on the chromosome maintenance [73]. 

The function of the RmuC-family protein containing an endonuclease domain remains unclear in this context [74]. It is annotated in phytoplasmas except for ‘*Ca*. P. mali' (ST4). 

Due to dispersed locations within the genome of ‘*Ca*. P. mali' it seems to be unlikely that the *ruv*- and *rec*-genes derived from a horizontal transfer. One may speculate that if this gene cluster is still functional as a unit. In comparison to *A. laidlawii*, similarities such as the lack of *ruvC* are obvious but *refN*, *recF*, and *refD* are encoded. 

Functional recombination machinery provides an advantage, if selection pressure induced by attacks from the environment or competition in the same ecological niche exists. This aspect is addressed in the statement of “living with instability” of phytoplasmas [49], so differences in coding capacity would enable response to such selection pressure. 

However, plasmid-derived proteins involved in replication such as Rep are encoded in asteris yellows phytoplasma chromosomes (PAM_667, AYWB_403).

### 3.4. DNA Repair and Degradation

UvrABCD proteins represent the genetic components of the nucleotide excision repair DNA repair system (ST5), which also is involved in SOS response. UvrA protein forms homodimers in the presence of ATP, binds to the DNA, and associates with UvrB. The two other units are formed by the nuclease UvrC and the helicase UvrD. They act jointly with the DNA ligase and complete the excision-repair process. The complete genetic repertoire is encoded in three phytoplasma genomes and *A. laidlawii* but is absent in ‘*Ca*. P. mali' with exception of UvrD, which is probably also involved in other reactions due to its helicase activity. 

All four phytoplasmas encode parts of the base excision repair pathway to repair certain types of premutagenic lesion (ST5). The genes *mutM* and *mutT* are present in all four phytoplasma genomes but additional genes of the *mut*-system such as the ATPase *mutLS* are missing. In contrast, they are present in *A. laidlawii*, which also carries *mutT* but lacks *mutM*. It is possible that other genes compensate the function of MutLS and enable the hydrolase and the glycolyse/lyase to function in combination with the other encoded proteins required. The annotated uracil-DNA glycolase would support the presence of such a basic system. 

The genetic repertoire for the nucleotide excision repair DNA for damaged oligonucleotides is encoded in ‘*Ca*. P. mali', while misincorporated bases cannot be corrected by the nucleotide excision repair system. This will result in uncorrected bases, which include the deamination leading to a C to A transversion and may affect the G + C content of ‘*Ca*. P. mali'. 

An incomplete restriction-modification system is encoded in strain OY-M but is absent in the closely related strain AY-WB and ‘*Ca*. P. australiense' and ‘*Ca*. P. mali'. This indicates that this system does not belong to the shared core set. It may thus be derived from a horizontal transfer.

### 3.5. DNA Modification and Structure

All phytoplasmas contain the bacterial core proteins encoding the type I topoisomerase TopA and the two gyrase subunits forming the type II topoisomerase (ST6). This protein content enables the phytoplasmas to relax supercoiled DNA by introducing single-stranded or double-stranded DNA breaks. 

The high copy number of methyltransferases and of the nucleotide-binding proteins is remarkable. The methylation protects the DNA, for example, from restriction by endonucleases, a strategy, which is used to protect the integrity of the chromosomal DNA and to decrease the number of integration events. The high number of nucleotide-binding proteins corresponds to the latter scenario. This is in agreement with the high number of copies of the integration host factor HimA, which is required in site-specific phage recombination [75] and is part of the complex transposons such as PMU1 [59]. The copy numbers of these genes differ even between closely related strains. Only a single copy of *himA* is present in ‘*Ca*. P. mali', corresponding to the putative single PMU [60].

## 4. Transcription

### 4.1. RNA Polymerase and Sigma Factors

RNA polymerase subunits for alpha, beta, beta', and omega chain are encoded in all 4 genomes (ST7). The sigma subunit is present with several paralogs, which is not unusual for bacterial genomes. The regulators RseABC and the sigma factors related to heat shock (RpoH), metabolic functions (RpoN), or stress (RpoS) were not identified. It is remarkable that the genomes of strain OY-M and of ‘*Ca*. P. australiense' encode a high number of *fliA* genes whereas ‘*Ca*. P. mali' encodes only a single gene. The small sigma factor FliA is known to be involved in transcriptions of operons involved in chemotaxis, motility and biofilm formation [76, 77]. However, there are no hints available on these abilities except for transcription in phytoplasmas.

### 4.2. Factors Affecting the RNA Polymerase

A common set of genes encoding proteins for transcription regulation and elongation is shared (ST8). It consists of the transcription elongation factor GreA and the N utilization substance protein involved in prevention and enhancement of transcription termination [78]. The transcription coupling factor Mfd [79] and the transcriptional termination factor Rho [80], like in *Mycoplasma* species, have not been identified in phytoplasmas.

### 4.3. Transcriptional Regulators

Only a sparse repertoire of transcriptional regulators is present in phytoplasmas. The negative regulator of class I heat shock proteins HrcA and putative cold shock proteins are encoded in all four phytoplasma genomes (ST9). Regulators of two-component systems were not annotated in these genomes.

## 5. Translation

### 5.1. rRNA-Operons

Two rRNA operons are encoded in the complete determined phytoplasma genomes. This applies for all phytoplasmas [81] and *A. laidlawii* (Acc. no. CP000896). The positions of the rRNA genes (Table 3) were recalculated using the actual version of the RNAmmer (v.1.2) software [82]. However, it should be taken in account that the prediction by RNAammer, which is based on HMM and BLAST, of start and stop position can differ by about 10 nt. A switch in strand preference of the rRNA operons with respect to the proposed *oriC* and *terC* is only present in the two aster yellows strains.

### 5.2. tRNA Synthetases

Aminoacyl tRNA synthetases catalyse the esterification of a specific amino acid or its precursor to one of all its compatible cognate tRNAs to form an aminoacyl-tRNA. They are essential for the translation process. 

In contrast to the other tRNA synthetases (ST10), the aspartyl/glutamyl-tRNA(Asn/Gln) amidotransferase subunits ABC (*gatABC*) are absent in all phytoplasma genomes and *A. laidlawii*. This absence separates the four phytoplasma genomes from other *Mycoplasma*, *Ureaplasma*, and *Mesoplasma* species.

### 5.3. Transfer RNAs

Transfer RNAs are essential for the transfer of a specific active amino acid to a growing polypeptide chain during translation. The complete set of tRNAs was recalculated for the four genomes by tRNAscan-SE v.1.23 using the bacterial model [83]. The qualitative endowment of tRNAs is shared by the phytoplasmas but there are quantitative differences. Most different is ‘*Ca*. P. australiense' by the presence of an additional gene for tRNA-Gln/His/Leu and only a single copy for tRNA-Val (ST11). Transfer RNA coding for the usage of selenocysteine was not identified in the four genomes and *A. laidlawii*.

### 5.4. tRNA/rRNA Modification Factors

All phytoplasmas share a similar set of modification factors (ST12), except for RimM, which was identified only in ‘*Ca*. P. mali'. It is also present in *A. laidlawii*. RimM is essential for the efficient processing of 16S rRNA in *E. coli* [84]. It has affinity for free ribosomal 30S subunits but not for 30S subunits in the 70S ribosomes. The absence in the other three phytoplasmas and *Mycoplasma* species indicates the distinct phylogenetic position of ‘*Ca*. P. mali' but also the higher relatedness to the acholeplasmas than the other three phytoplasmas.

### 5.5. Translation Factors

All four genomes share a common set of translation factors including LepA (elongation factor 4) (ST13). LepA is a ribosomal back translocase related to EF-G and EF-Tu and is supposed to recognize ribosomes after a defective translocation reaction. This reaction allows EF-G to translocate the tRNAs correctly [85]. 

PrfA and PrfB mediate translation termination. The presence of these proteins is in accordance with the observation that not only the four fully sequenced phytoplasma genomes but all phytoplasmas use the bacterial code. PrfB is absent in *Mycoplasma* species, which translate UGA as tryptophan rather than using it as a stop codon [86].

### 5.6. Ribosomal Proteins and Modifying Factors

The environment for genes encoding ribosomal proteins consisting of 32 large subunits and 20 small subunits was identified in all phytoplasma genomes (ST14), in addition to some modifying enzymes (ST15). The genes encoding the large subunit protein RplY (L25) and the small subunit protein RpsA (S1) were not identified. However, RpsA was identified in *A. laidlawii*. 

Some genes seem to be absent in phytoplasmas such as the ribosomal protein L11 methyltransferase (PrmA) or FtsJ. They also are absent in other mollicutes. The glutathione synthetase RimK present in *M. pneumoniae* and *M. genitalium* is also absent in all phytoplasmas. 50S ribosomal stability factor encoded by the subunits *engABCD* is present in all phytoplasmas.

### 5.7. mRNA Degradation

All four genomes encode proteins similar to YkqC from *Bacillus licheniformis* (YP_078844.2) encoding the essential RNase J1/J2 involved in mRNA degradation (ST16).

### 5.8. Heat Shock Proteins and Chaperons

The four phytoplasmas share a basic gene set containing the Hsp70-type (*dnaK*, *grpE*), the Hsp60-type (*groEL*, *groES*) chaperone system [87], the chaperone *dnaJ*, and the trigger factor (ST17). The small heat shock protein IbpA (Hsp20) that binds to aggregated proteins and is present in other phytoplasmas [88] was not identified in ‘*Ca*. P. mali'. 

Other encoded cytosolic proteases such as Lon-protease that hydrolyses ATP and unfolds bound substrates are encoded in all four phytoplasmas. These proteins are involved in various cellular activities [89].

## 6. Cell Envelope and Cell Division

### 6.1. Proteins Involved in N-Acetylglucosamine and Murein Biosynthesis

Proteins involved in N-acetylglucosamine and murein biosynthesis have not been identified in phytoplasma genomes. The absence of these genes is in accordance with the lack of a cell wall. However, all four genomes encode MraW (ST19), which is a S-adenosyl-dependent methyltransferase also described to show activity toward substrates associated with membrane components [90].

### 6.2. Proteins Involved in Lipopolysaccharide Synthesis

Only *rfaG* encoding for core functionality in lipopolysaccharide (LPS) synthesis was identified in ‘*Ca*. P. asteris' strain OY-M and ‘*Ca*. P. mali'. RgaG acts as a UDP-glucose: (heptosyl) LPS *α*-1,3-glucosyltransferase in *E. coli* [91] releasing UDP and H^+^. This enzyme is involved in lipid A-core biosynthesis (ST20). Other proteins of this pathway were not identified.

### 6.3. Cell Division Proteins and Regulators

In enterobacter, cell division is mainly mediated by *fts* and *min* genes that determine cell shape. In phytoplasmas cell division proteins FtsY and FtsH were identified. Two additional genes associated with cell division were detected (ST21). The impact of the glucose-inhibited division (Gid) proteins encoded by phytoplasmas on the regulation of the cell division processes remains unclear. GidA is reported to be involved in 5-carboxymethylaminomethyl modification of the wobble uridine base in some tRNAs [92, 93] whereas GidB, which is missing in ‘*Ca*. P. mali', is suggested to act as S-adenosyl-L-methionine- (SAM-) dependent methyltransferase [94]. Both genes are encoded in the majority of bacterial genomes and also encoded in the genome of *A. laidlawii*. However, it was shown by mutagenesis of *M. genitalium* and *M. pneumoniae* that GidB is not essential [95]. It was suggested that the GidB of *E. coli* is specific for sterol and/or lipid substrates, but it also seems to be possible that GidB is specific for nucleic acids [96].

## 7. Membrane Proteins, Secretion, and Transport

### 7.1. Porins and Outer Membrane Proteins

Phytoplasma membrane proteins are in direct contact with their environment due to the lack of a cell wall. Membrane proteins such as Vmp1 show a wide diversity in accordance with their importance for phytoplasmas [97]. A subset of the membrane proteins are the abundant immunodominant proteins, which also show a high diversity [98]. Proteins such as the antigenic membrane protein AmP interact with the insect cell microfilaments and contribute to the insect vector specifity [99]. They are under positive selection pressure [100]. AmP is encoded in the genomes of the two ‘*Ca*. P. asteris' strains. A putative homolog is also present in ‘*Ca*. P. australiense', while ‘*Ca*. P. mali' encodes the immunodominant protein ImP (ST22) [101]. However, *imp* and *amp* were also identified in ‘*Ca*. P. asteris' strain OY-W [102]. 

These proteins represent only a small part of the predicted membrane proteins that are characterized by at least one transmembrane (TM) helix (ST23). Integral membrane proteins and secreted proteins have been efficiently predicted by Phobius [103] from the annotated proteins in all four genomes. The ‘*Ca*. P. asteris' strains OY-M and AY-WB carry 184 and 169 proteins containing at least one TM domain but no signal peptide (SP). The large chromosome of ‘*Ca*. P. australiense' encodes 181 of such proteins and ‘*Ca*. P. mali' with the shortest chromosome 145. Some membrane proteins carry a SP in addition that indicates a cleavage during protein translocation [98]. A low number of these proteins have been predicted in phytoplasmas: 12 in ‘*Ca*. P. asteris' strain OY-M, 9 in strain AY-WB, 10 in ‘*Ca*. P. australiense', and 5 in ‘*Ca*. P. mali' (ST24). 

The majority of membrane-associated proteins involved in transport and metabolism will be treated within the following sections.

### 7.2. *Sec*-Dependent Pathway (General Signal-Dependent Export Pathway)

The *sec*-dependent pathway represents the best-characterized secretion system of phytoplasmas. The four phytoplasma genomes share a common gene set of *sec*-genes. (ST25). Genes encoding proteins of the general secretion pathways (T2SS) were not identified and also the twin arginine pathway for the secretion of folded proteins and their cofactors is absent. This can be explained by the absence of a second outer membrane. Other secretion pathways of phytopathogenic bacteria such as the type III secretion system (T3SS), T4SS, or pili have not been identified [104]. There is weak indication for a deduced IcmE-like membrane protein involved in a T4SS (*orf552* in ‘*Ca*. P. australiense') [50] but further analysis is needed. 

**Figure 5:**
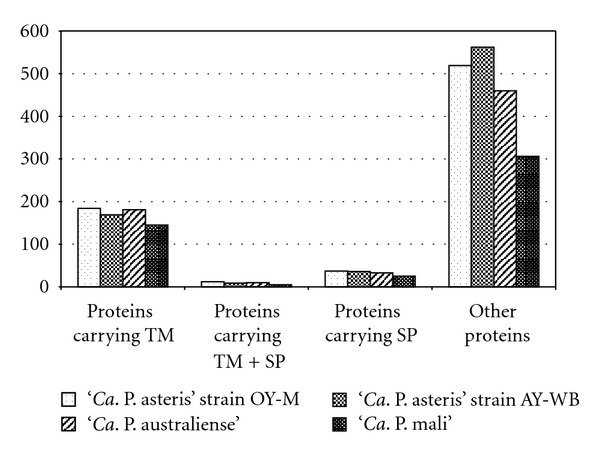
Phobius predictions of the four phytoplasma genomes. Abbreviations: TM: transmembrane helices; SP: signal peptide.

Secreted phytoplasma proteins may directly interact with their hosts and may in this way manipulate or weaken the plant host or insect vector without a needled system as provided by T3SS. Examples for the phytoplasmas are the secreted proteins tengu and SAP11 of ‘*Ca*. P. asteris' strains OY-M and AY-WB, respectively [105]. For tengu it has been shown that the protein expression in *Arabidopsis thaliana* results in dwarfism [106]. The *sec*-dependent pathway is encoded in all four genomes by *secYEG* forming the integral membrane pore complex (Figure 5) and completed by *secA* and *yidC* [107]. YidC mediates the membrane insertion/assembly of the inner membrane proteins [108]. 

Genes encoding SecB, SecD/F, SecG, and YajC were not identified in the four genomes (ST25). GroEL may fulfil the function of the chaperone SecB. The effect of the absence of bifunctional SecD/F protein remains unclear due to the lack of information on their exact function. They might act to facilitate the protein export [109, 110]. The gene encoding the integral membrane protein *secG* is present in *A. laidlawii* but absent in phytoplasmas. A similar situation is present for YajC, which is supposed to stabilize the insertion of the SecA-preprotein complex in other bacteria. 

The signal peptide mechanism seems to be similar to *E. coli*, and prediction software such as SignalP [111] or PSORT developed for Gram-negative bacteria [112] can be applied for the prediction of proteins secreted by the *sec*-dependent pathway [107] and other software such as Phobius [103] limiting the analysis on proteins carrying a signal peptide but no transmembrane regions (Figure 5). The ‘*Ca*. P. asteris' strains OY-M and AY-WB carry 37 and 36 predicted secreted proteins containing a SP, while ‘*Ca*. P. australiense' encodes 33 and ‘*Ca*. P. mali' 25 (ST26).

### 7.3. Signal Recognition Particle (SRP) Pathway

The SRP is a ribonucleoprotein, which was identified in all organisms. It acts in targeting translating ribosomes to the SecYEG translocation complex for cotranslational protein translocation over the membrane [113]. The SRP complex consists of the RNA component (4.5S RNA, *ffs* gene product) and the protein component (the *ffh* gene product). This complex is required for targeting some integral membrane proteins to the membrane for cotranslational integration [114]. Additional proteins are required for the functional translocation system. They comprise YidC (see above), which is bound to the SecYEG translocation complex and FtsY. The latter serves as docking protein for the SRP-ribosome complex synthesising the nascent peptide [115]. 

All units forming this protein complex were encoded in the four phytoplasmas (ST27) except the *secG* and the *ffs* gene for 4.5S RNA, which were also not identified in acholeplasmas. However, it is difficult to identify 4.5S RNA that is encoded by eubacteria, archebacteria, and *Mycoplasma* species [116].

### 7.4. ABC Transporters and Symporters

**Figure 6:**
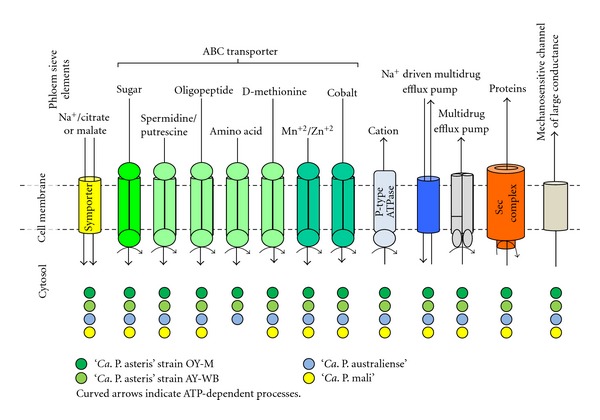
Symporter, transporter, ion translocation, and secretion.

All four phytoplasmas depend on the uptake of essential compounds from their hosts (Figure 6) [48]. They encode a common core set of ABC transporters (ST28). This includes the clustered spermidine/putrescine transport system (*potABCD*), a Mn/Zn transport system (*znuACB*), sugar transport system (assigned as *malEGFK*), a dipeptide/oligopeptide transport system (assigned as *dppCBADF*), and a methionine transport system (*metNQI*). An additional amino acid transport system (*artQPIM*) is encoded in OY-M (probably duplicated), AY-WB, and ‘*Ca*. P. australiense' but is absent in ‘*Ca*. P. mali'. The genes for *znuACB* are present in two copies in ‘*Ca*. P. mali' due to the location in the terminal inverted repeats. 

The template of the sugar transport system remains unclear. The uptake of maltose, trehalose, sucrose, and palatinose was suggested, and sucrose appears to be most likely [50, 117] with respect to the presence in the phloem and the corresponding protein machinery. *Spiroplasma citri*, another phloem colonizing parasite, is able to utilize glucose, fructose, and trehalose [118]. 

The subunits in phytoplasmas show similarities to maltose/maltodextrin transporters but also to glycerol-3-phosphate transporters (*ugpBEAC*). Both protein sets are highly homologous and might be functionally exchangeable, but show differences with respect to the growth substrates [119]. The import of glycerol-3-phosphate would also offer a phosphate source for the phytoplasmas. 

The phytoplasma dipeptide/oligopeptide transport system shows sequence differences in the substrate-binding unit that may indicate differences in substrate specifity. 

In addition, several incomplete ABC transporters (ST29) are present in phytoplasmas. Subunits of the above mentioned transporters of dipeptide/oligopeptide transporters, amino acids, and cobalt are present in the genome. Incomplete transporter units might be completed by subunits of other peptide and amino acid transporters. The functionality of cobalt import system remains unclear because the periplasmatic subunit was not identified. However, the permease (*cbiQ*) and ATPase subunits (*cbiO*) are present in all phytoplasma genomes. 

A putative thiamine transporter subunit is encoded in all four phytoplasma genomes (ST30). The corresponding peptide sequences were assigned by InterPro [120] to the YuaJ-family (IPR012651). Many YuaJ-family members have been assigned as ATP-independent thiamine transporter, which are involved in regulation of the thiamine pyrophosphate (TPP) concentration [121]. The import is probably proton coupled. Thiamine is phosphorylated at the membrane by thiamine kinase (ThiK) in *Bacillus cereus* [122]. ThiK has not yet been identified in mollicute genomes. This raises the question if thiamine is imported or if it is an already phosphorylated substrate. The thiamine monophosphate kinase ThiL (syn. ThiJ) is encoded in the four phytoplasma genomes (PAM_137, AYWB_584, PAa_0495, ATP_00237). This finding supports the idea that a phosphorylated thiamine substrate or an unassigned protein has to fulfil the function of ThiK in phytoplasmas. 

Beside the ABC transporters, malate or citrate/Na^+^ symporters are encoded in all four genomes that may provide an important carbon source [49] (ST31). 

Several other membrane-located proteins are involved in transport in all four genomes and belong to the core gene repertoire of phytoplasmas (ST32). They contain a multidrug efflux pump such as MdlAB and the Na^+^-driven multidrug efflux pump NorM. The genes assigned as *norM* differ in sequence similarity. The NorM protein from ‘*Ca*. P. mali' has only one ortholog in ‘*Ca*. P. australiense' (PAa_0171). 

Another common feature is the P-type ATPases exporting cations such as magnesium, calcium, and cadmium (ST33). Notably, mutants deficient in the single P-type ATPase encoded in *Spiroplasma citri* are affected in their growth capacity [123]. 

Finally, the regulation of the osmotic pressure by opening the membrane lipid bilayer to prevent cell disruption and death [124] is given by the mechanosensitive channel formed by the MscL protein in phytoplasmas (ST34). 

## 8. Metabolism

### 8.1. Carbohydrate Metabolism Glycolysis

Embden-Meyerhof-Parnas pathway was suggested to be the major energy-yielding pathway in phytoplasmas [48] despite the apparent lack of hexokinase (glucose phosphorylating) and a sugar-specific phosphotransferase system (PTS) mediating a phosphorylated hexose to enter glycolysis. One promising candidate is the glucosyltransferase GtfA, which was predicted in ‘*Ca*. P. australiense' first. The assignment of the deduced protein sequence and the ortholog of OY-M were confirmed by InterPro (IPR022527 sucrose phosphorylase, GftA). The GtfA, which is probably better described as disaccharide glucosyltransferase or sucrose phosphorylase, allows the formation of *α*-D-glucose-1-phosphate from phosphate and sucrose [125], which is often a predominant sugar in the phloem. *α*-D-glucose-1-phosphate is the entry compound of glycolysis. GtfA may compensate the absence of a hexokinase and PTS system. However, GtfA or a similar phosphorylase are not a general trait of phytoplasmas, since they are absent in the genomes of closely related AY-WB and in ‘*Ca*. P. mali'. Thus, the observed differences in glycolysis among phytoplasmas may arise from genome plasticity as suggested by the close proximity of prophage-related elements and *gtfA* in ‘*Ca*. P. australiense'. 

Theoretically, the uptake of phosphorylated hexoses would overcome this problem. Candidates for such sugar phosphates are trehalose-6-phosphate, sucrose-6-phosphate, and *β*-D-fructose-6-phosphate. The transporter complements of the phytoplasmas may contain uptake systems for importing these phosphorylated di- and monosaccharides from the environment. For example trehalose-6-phosphate has to be monomerized prior to entry into the glycolysis. The breakdown would occur within the phytoplasmas mediated by hydrolases/phosphatases. At least one phosphatase each of subfamily IIIa and of subfamily IIb is encoded within the four phytoplasma genomes (ST35). These phosphatases are poorly characterized so far. A single copy of the IIIA subfamily of the haloacid dehalogenase (HAD) superfamily of hydrolases representing hypothetical proteins is encoded in each phytoplasma genome. Most characterised members of this subfamily and of the HAD superfamily are phosphatases. This protein family consists of sequences from fungi, plants, cyanobacteria, Gram-positive bacteria, and *Deinococcus* (according to IPR010021 entry). 

Functional interpretation of the second hydrolase of the Had superfamily hydrolase subfamily IIb may be more straight forward. Members of the Had superfamily hydrolase subfamily IIb are encoded by at least one gene (‘*Ca*. P. mali') in each genome. They encompass trehalose-6-phosphatase, plant and cyanobacterial sucrose phosphatise, and a closely related group of bacterial and archaeal orthologs, eukaryotic phosphomannomutase (according to IPR006379 entry). If these proteins function as trehalose-6-phosphatase, phytoplasmas could use *α*, *α*-trehalose-6-phosphate, and phosphate to produce glucose-6-phosphate and *β*-D-glucose-1-phosphate, which are the entry molecules of glycolysis. 

If sucrose-6-phosphate is used as a substrate, sucrose-6-phosphatase may generate glucose-6-phosphate and fructose [126]. The utilization of trehalose-6-phosphate and/or sucrose-6-phosphate appears to be likely due to the phosphoglucose isomerase encoded in all four genomes. This step would be unnecessary, if fructose-6-phosphate is available. However, it should be considered that only trace amounts of trehalose-6-phosphate are present in higher plants on average. The impact of a phytoplasma infection on plant metabolism cannot be estimated so far, but trehalose-6-phosphate is a signaling molecule in plants with strong regulatory effects on metabolism, growth, and development [127–129]. The sucrose-6-phosphate concentration in the phloem is unclear. It is an interesting scenario that sucrose and trehalose compounds could be utilized depending on their availability in phloem and hemolymph. 

**Figure 7:**
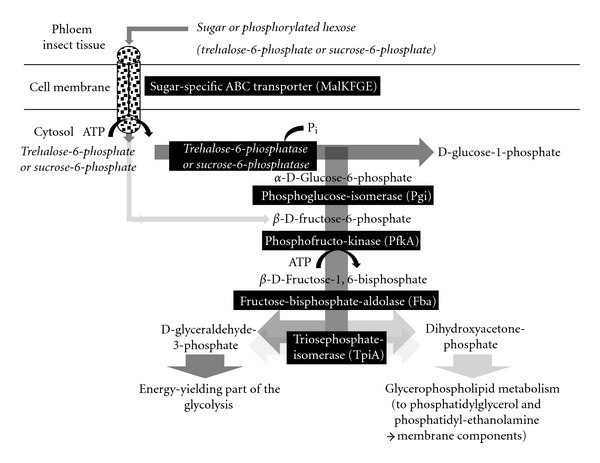
The sugar uptake and upper part of the glycolysis (energy investment) including trehalose-6-phosphate and *β*-D-fructose-6-phosphate as suggested entry substrates (steps indicated in italics).

The general upper part of the glycolysis (energy demanding) is encoded within all four phytoplasma genomes [20, 48–50] starting with *α*-D-glucose-6-phosphate converted to *β*-D-fructose-6-phosphate by phosphoglucose-isomerase (Pgi) and ATP-dependent formation of two molecules *β*-D-fructose-1,6-bisphosphate by phosphofructose kinase (PfkA). Subsequently, fructose-bisphosphate-aldolase (Fba) catalyses the formation of D-glyceraldehyde-3-phosphate and dihydroxyacetone-phosphate. The latter is suggested to enter glycerophospholipid metabolism, while D-glyceraldehyde-3-phosphate is channelled into the energy yielding part of the glycolysis (Figure 7). The interconversion of these C_3_-intermediates is performed by triosephosphate isomerase (TpiA). 

Dihydroxyacetone phosphate can also be generated by a conserved kinase related to dihydroxyacetone kinase (DhaK). However, it remains unclear if this kinase can also act in the opposite direction as a transferase. 

**Figure 8:**
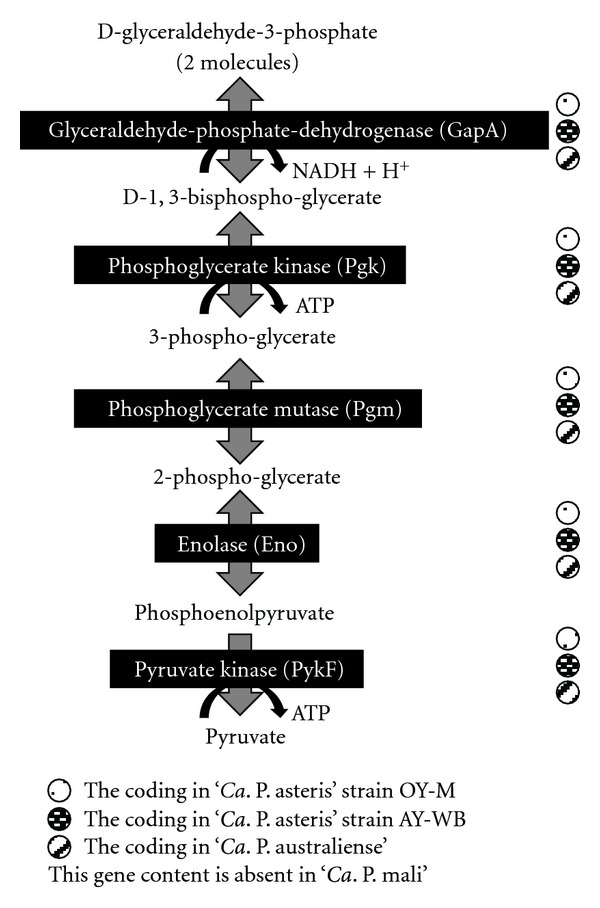
The energy-yielding part of the glycolysis encoded in the asteris strains and ‘*Ca*. P. australiense' but absent in ‘*Ca*. P. mali'.

 Except for ‘*Ca*. P. mali' [20] the protein components of the lower part of glycolysis (energy yielding) from D-glyceraldehyde-3-phosphate to pyruvate are encoded in the analyzed phytoplasma genomes (ST35): glycerinaldehyd-phosphate dehydrogenase, phosphoglycerate kinase, and mutase, enolase, and pyruvate kinase (Figure 8). Notably, gluconeogenic phosphoenolpyruvate synthase (PpsA) is lacking in all four phytoplasmas (Figure 8). Other strategies to obtain host-derived ATP such as ATP/ADP translocase known from *Chlamydia* species [20, 48, 130] and also the arginine dihydrolase pathway [131] have not been identified in phytoplasmas. 

### 8.2. An Alternative Energy-Yielding Pathway Deduced from the Genome Sequences

**Figure 9:**
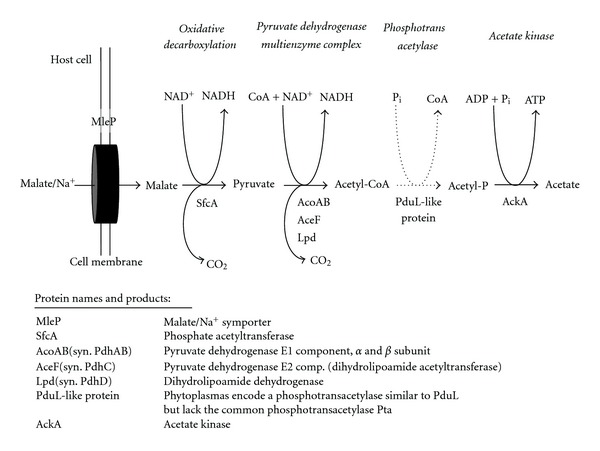
Proposed energy-yielding pathway from malate to acetate.

Carbohydrate metabolism is one of the most important physiological traits of the phytoplasmas. Alternative pathways have to be considered that could compensate for the lack of a complete glycolysis in ‘*Ca*. P. mali' (Figure 9, ST36). A pathway for malate conversion to acetate is potentially encoded in all four genomes. Uptake of malate is enabled by the symporter MleP [20, 48]. Malate can be oxidatively decarboxylated to pyruvate by the malic enzyme ScfA. Pyruvate would then also be oxidatively decarboxylated to acetyl-CoA by the pyruvate dehydrogenase multienzyme complex. 

In the case of ‘*Ca*. P. mali' pyruvate might be generated by an additional way. Here, an aldolase (Eda) is predicted, serving two possible functions. A 4-hydroxy-2-oxoglutarate aldolase (EC: 4.1.3.16) (KHG-aldolase) would catalyze the interconversion of 4-hydroxy-2-oxoglutarate into pyruvate and glyoxylate. Phospho-2-dehydro-3-deoxygluconate aldolase (EC: 4.1.2.14) (KDPG-aldolase) would catalyse the interconversion of 6-phospho-2-dehydro-3-deoxy-D-gluconate into pyruvate and glyceraldehyde-3-phosphate. In both cases, pyruvate would be formed independently from glycolysis. 

It is also notable that the gene set necessary for the generation of coenzyme A (CoA) is only partially annotated in phytoplasmas. In *E. coli*, CoA is synthesized from (R)-pantothenate. This requires involvement of 4′-phosphopantothenoylcysteine decarboxylase and phosphopantothenoylcysteine synthetase (fused in *E. coli*, CoaBC), the phosphopantetheine adenylyltransferase (CoaD), and the dephosphocoenzyme A kinase (CoaE). CoaBC was identified in some *Mollicutes*, while CoaD is annotated in the majority of phytoplasmas and *A. laidlawii*. While genes encoding CoaBC and CoaD were not identified in phytoplasmas, CoaE (annotated as formamidopyrimidine-DNA glycosylase) is encoded in all four genomes. The intermediate dephospho-CoA is the substrate of CoaE. This finding supports the possibility that at least the last steps in CoA-biosynthesis are performed by the phytoplasmas, but it remains unclear whether alternative reactions for the other steps in the pathway are encoded in the genomes. 

Hydrogen peroxide (H_2_O_2_) could be formed by HcaD, oxidizing NADH. H_2_O_2_ may also be generated by the encoded superoxide dismutase and represent a potential virulence factor, as known from *M. pneumoniae* and also encoded in *A. laidlawii* [132–134]. 

Acetyl CoA can be converted to acetyl phosphate phosphotransacetylase (Pta) in many mycoplasmas. Subsequently, acetyl phosphate can be transformed by acetate kinase (AckA) to acetate and ATP (Figure 9). However, Pta, which is encoded on many mycoplasma chromosomes close to *ackA*, is absent from all four phytoplasma genomes [14, 20]. Lacking Pta could be substituted by another phosphotransacetylase (PduL) described for *Salmonella enterica *subsp. *enterica* serovar typhimurium (Acc. no. AAD39011), since it is encoded in all four phytoplasma chromosomes. This assignment of Pdul is in accordance with the kinetic parameter with a KM value of 0.97 mM for acetyl phosphate and a *V*_max⁡_ of 13.4 *μ*M/min/mg enzyme with acetyl phosphate as substrate (according to Uniprot entry Q9XDN5). 

The assignment of the phosphotransacetylase as propanediol utilisation-like protein PduL in phytoplasmas remains ambiguous. Propanediol is produced by fermentation of the common plant sugars rhamnose and fucose. In *S. enterica*, PduL is part of the *pdu*-operon and involved in the coenzyme-B_12_-dependent degradation of 1,2-propanediol [135–137]. It seems to be likely that in phytoplasmas, phosphotransacylase PduL does not function in propanediol degradation, since other genes of this pathway (e.g., *pduCDE*, *pduQ*, *pduP*, and *pduW*) are absent in phytoplasmas and *A. laidlawii* (except for *pduL*). Thus, PduL could use acetyl-CoA instead of propionyl-CoA as substrate. 

### 8.3. Associated Processes

#### 8.3.1. Proteins Involved in NAD Synthesis

All four phytoplasma chromosomes encode the glutamine-dependent NAD^+^ synthetase NadE (ST49). In this ATP-dependent process nicotinate adenine dinucleotide (deamido-NAD^+^), L-glutamine, and H_2_O are used to form L-glutamate, AMP, diphosphate, H^+^, and NAD^+^. Other proteins involved in the generation of NAD^+^ were not identified in the phytoplasma genomes. In contrast, *A. laidlawii* contains NadD and PncB allowing the formation of NAD^+^ from nicotinate. It appears likely that phytoplasmas import deamido-NAD^+^ or a precursor from their environment.

#### 8.3.2. Proteins Involved in Oxygen Detoxification

All four genomes encode a superoxide dismutase (SOD), which converts O_2_^−^ to the less toxic H_2_O_2_ (ST40). SOD activity is documented for several *Mollicutes* including the genera *Mycoplasma*, *Ureaplasma*, and *Acholeplasma* [138]. SOD requires metal cations such as copper, manganese, iron, or nickel. Prokaryotes were assigned to form cytoplasmatic MnSOD and/or FeSOD [139], but for *M. hyopneumoniae* the production of Cu/ZnSOD was shown [140]. The formation of MnSOD is reported for the acholeplasmas [141]. The catalase dismutase responsible for the conversion of H_2_O_2_ to nontoxic products was not identified in phytoplasmas so far. This is also reported for several other mycoplasmas and may contribute to virulence of these organisms by release of reactive H_2_O_2_ [142]. It remains unclear, whether the release of H_2_O_2_ will weaken or damage the plant host upon presence of phytoplasmas inside the sieve elements.

#### 8.3.3. Lipoyl-Protein Ligase

Lipoic acid derivates act as cofactors in enzymatic systems, such as the pyruvate dehydrogenase [143] in the phytoplasmas. All four chromosomes encode the ATP-dependent lipoyl-protein ligase LplA (ST50), which preferentially utilizes imported lipoate to form lipoyl adenylate or an octanylated protein from octanylate [144].

## 9. Purine and Pyrimidine Synthesis

**Figure 10:**
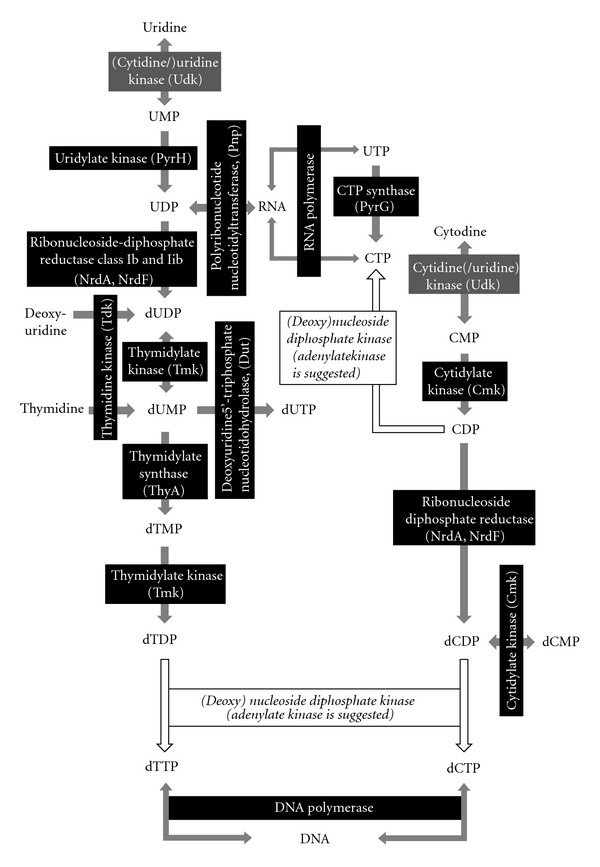
Principle of pyrimidine metabolism in phytoplasmas.

Purine and pyrimidine metabolism in *Mollicutes* was reviewed recently [145]. Therefore, the following focuses on the genetic key elements or aspects of general interest for the phytoplasmas (ST42). Since phytoplasmas possess no genetic repertoire for *de novo* synthesis of purine or pyrimidine bases [48], they depend like many other mollicutes on environmentally derived nucleotide precursors [145]. However, nucleobase or nucleoside transporters were not identified in phytoplasmas so far [48], but it was suggested for mycoplasmas that the limited repertoire of transporters could be tuned to a wider variety of substrates [146]. Membrane-associated nucleases are suggested for the nucleotide precursor uptake and were identified in 20 mycoplasma species [147]. Membrane nucleases such as MnuA of *M. pulmonis* [147] were characterized, and putative orthologs were identified in *M. hyopneumoniae*, *M. gallisepticum*, *M. pneumonia*, *M. penetrans*, and *U. urealyticum *[145]. However, it was not possible to identify candidates in phytoplasmas so far. The uptake and incorporation of dNMP without prior dephosphorylation was shown for *M. mycoides* subsp. *mycoides* [148]. The presence of such a strategy in phytoplasmas is important for nucleotide and energy metabolism. However, all four genomes encode common pathways for purine and pyrimidine synthesis with one exception (Figure 10). The usage of uridine and cytodine in the ‘*Ca*. P. australiense' and ‘*Ca*. P. mali' remains questionable, because the cytidine/uridine kinase (Udk) mediating the formation of CMP/UMP is absent in both chromosomes. However, it is present in *A. laidlawii* (YP_001620379). 

**Figure 11:**
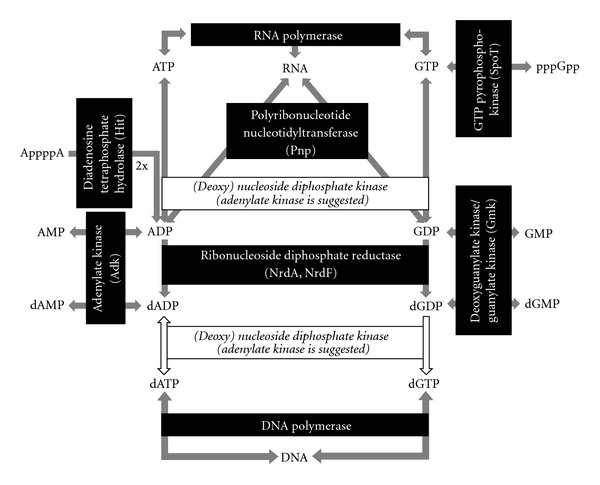
Principle of purine metabolism in phytoplasmas.

AMP and dAMP as well as GMP and dGMP represent the entry points into purine metabolism in all four genomes (Figure 11). The (deoxy)nucleoside diphosphate kinase (Ndk) is absent in the four phytoplasma genomes. This was also observed with many other mycoplasmas, the 6-phosphofructo-, phosphoglycerate-, pyruvate-, and acetate-kinases which could use other ribo- and deoxyribopurine and pyrimidine NDPs and NTPs besides ADP/ATP [146]. Similarly it was shown for *Mycobacterium tuberculosis* that the adenylate kinase (AdK) acts as general (deoxy)nucleoside diphosphate kinase (NdK) [149], a scenario that was also proposed for mycoplasmas [145]. 

CMP and CTP are also produced as byproducts within the phospholipid metabolism. However, it should be also noted that such function of the Adk might also result in an imbalance of the nucleotide pool and in the low G + C content of phytoplasma chromosomes in evolution. Notably, ‘*Ca*. P. mali' with the lowest G + C content encodes one *adk* on each terminal repeat of the chromosome.

## 10. Miscellaneous

### 10.1. Lipid Synthesis

**Figure 12:**
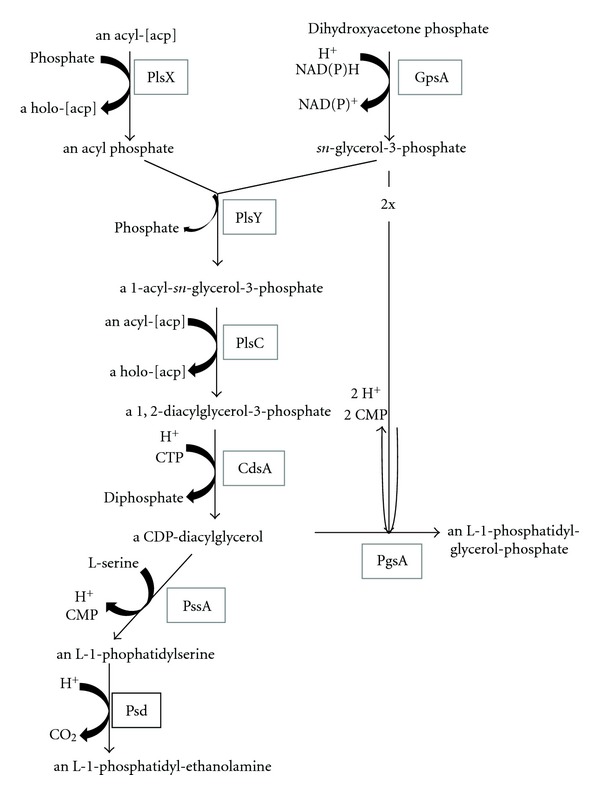
Key elements of the lipid metabolism.

All four phytoplasma genomes encode a common biosynthetic pathway for essential phospholipids (Figure 12, ST44), that is, for the formation of CDP-diacylglycerol from an acyl phosphate and dihydroxyacetone phosphate. Moreover, the genetic modules for the generation of L-1-phosphatidyl-glycerol and L-1-phosphatidyl-ethanolamine are present. The potential to form cardiolipin from L-1-phosphatidyl-glycerol remains unclear, since a cardiolipin synthase is apparently not encoded.

### 10.2. Proteolysis

Diverse proteases involved in the breakdown of proteins are encoded in the phytoplasma genomes. Several encoded peptidases enable the degradation of essential imported peptides and regulation of intracellular products. 

A remarkable number of zinc-dependent proteases are predicted from the phytoplasma genomes. For example the four genomes share the protease PmbA and Zn-dependent protease TldD (ST38), and the exact role in phytoplasmas remains elusive. 

The annotated zinc-dependent HflB metalloproteases may play a prominent role in the ‘*Ca*. P. asteris', due to the high number of paralogs: 19 in strain OY-M and 10 in strain AY-WB. High numbers of paralogs are also present in ‘*Ca*. P. australiense' [6] and ‘*Ca*. P. mali' [11]. These *hflB* gene products show differences in the encoded peptide length and domain composition. In *E. coli*, HflB is affiliated with multiple cellular functions, including a putative involvement in lysogeny of lambda phage [150]. Thus, one may speculate that the high number of *hflB* genes resulted from a high pressure of phage attacks. Studies on the various *hflB* genes and their presence within the genomes of differently virulent (mild to severe) strains are on the way.

### 10.3. Amino Acid Synthesis and Modification

It was not possible to detect proteins involved in the synthesis of amino acids, except for *S*-adenosyl-L-methionine (MetK), L-asparagine (AsnB), and L-ornithine (ArgE) (ST41, supplementary Figure 1 (SF1)). However, these enzymes are neither encoded in all four genomes nor do they enable the phytoplasmas to synthesise any amino acid [48]. Thus, all necessary amino acids or peptides have to be imported.

### 10.4. Riboflavin Synthesis

The only detected putative protein involved in riboflavin syntheses was ATP-dependent riboflavin kinase (ST45) in strain OY-M.

### 10.5. Folate Synthesis

**Figure 13:**
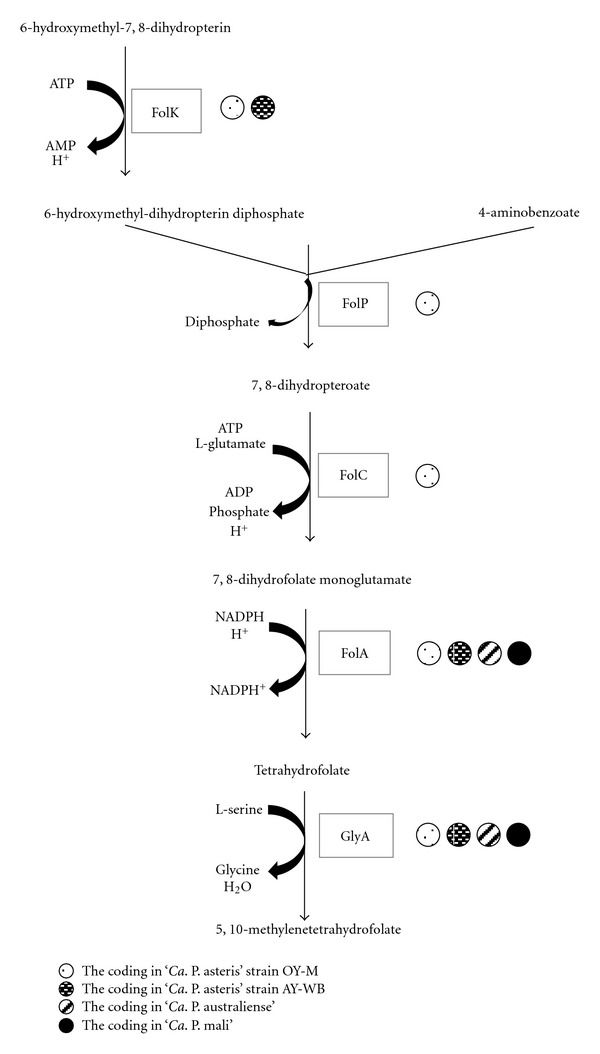
Folate synthesis.

Folic acid (or vitamin B9) is a precursor of the coenzyme tetrahydrofolate (THF). All four phytoplasma genomes (ST46) encode dihydrofolate reductase (FolA), which uses 7,8-dihydrofolate monoglutamate and NADPH + H^+^ for the generation of THF (Figure 13). Only strain OY-M encodes a gene set, which may allow generating THF from other precursors [49]. In the clover phyllody phytoplasma, genes assigned as *folP* and *folK* encode frameshifts and represent pseudogenes [151]. It remains unclear how phytoplasmas obtain 7,8-dihydrofolate monoglutamate and how they process 5,10-methylenetetrahydrofolate. The incomplete folate synthesis may indicate the loss of this genetic modules and folate-dependance on the host. This may influence the host because folate is also involved in photorespiration, amino acid metabolism, and chloroplastic protein biosynthesis in plants [152].

### 10.6. Thiamine Synthesis

All four phytoplasma genomes encode the ATP-dependent thiamine-phosphate phosphotransferase ThiJ (syn. ThiL) (ST47), which forms the coenzyme thiamine pyrophosphate (syn. thiamine diphosphate) from thiamine phosphate. ThiI, which is encoded in the chromosomes except for ‘*Ca*. P. mali', is required for thiazole synthesis in the thiamine biosynthesis pathway [153].

### 10.7. Pyridoxal phosphate Synthesis

The kinase PdxK of the vitamin B6 metabolism is only encoded in the genome of ‘*Ca*. P. asteris' OY-M (ST48). This ATP-dependent kinase catalyses the formation of pyridoxine-5′-phosphate from pyridoxal, pyroxidine, and pyridoxamine.

### 10.8. Iron-Sulfur Cluster Biosynthesis

NifU-like proteins are predicted to occur in all four phytoplasmas (ST39). Proteins such as NifS and NifU are required for the formation of metalloclusters of nitrogenase in *Azotobacter vinelandii* and the maturation of other FeS proteins. No further genes associated with nitrogen fixation were found in the phytoplasma genomes. Notably, *nifU*-like genes are also encoded in genomes of other organisms lacking the ability to fix nitrogen [154].

### 10.9. Acyl Carrier Protein Metabolism

Two proteins assigned to the acyl carrier metabolism are encoded in all four genomes (ST43): the acyl carrier protein AcpP, which is involved in the biosynthesis of fatty acids and membrane-derived oligosaccharides in *E. coli *[155] and the holo-[ACP] synthase (AcpS) involved in carrier formation in lipid synthesis [156].

### 10.10. Phosphate Metabolism

A set of six proteins involved in the metabolism of phosphorous compounds is encoded in all four genomes (ST51). The inorganic pyrophosphatases (Ppa) catalyse the hydrolysis of inorganic pyrophosphate (PPi) to two orthophosphates (Pi), regulating the PPi pool which is replenished by various metabolic processes [157]. Additional predicted proteins related to phosphate metabolism are phosphohydrolases, metallophosphoesterases, and lysophospholipases.

### 10.11. *beta*-Glucanase

Several genes with a weak catalytic or unclear pathway assignment are shared between some of the four phytoplasmas. An example is the predicted endoglucanase FrvX (M42 peptidase family). Considering that endo-1,4-beta-glucanase proteins in *Arabidopsis thaliana* are associated with plant growth (in particular elongation), xylem development, and cell wall thickening [158] the phytoplasma FrvX protein could potentially contribute to virulence (ST52).

## 11. Outlook

Future research has to experimentally validate the functions of the key proteins in phytoplasmas. The labelling of substrates and heterologous expression in cultivable hosts are possible strategies as long as axenic cultures of phytoplasmas are unavailable. Beside this basic molecular research at least two further fields of research have to be developed for phytoplasmas. 

First, a ‘*Ca*. Phytoplasma species' with a less reduced genome and/or a ‘*Ca*. Phytoplasma species' closer affiliating with the phylogenetic separation of *Acholeplasma* and ‘*Ca*. Phytoplasma' has to be identified. Studies on such an ancestor will provide insights into the evolution of the genomes and the host relationship. 

Second, gene expression data from a strain with complete and a host with at least a draft genome sequence are needed. To date, most insights were obtained from pathogens, but these data need to be integrated with the situation in the healthy plant, to learn more about the overall plant response.

## Supplementary Material

Supplementary tables provide information on the gene content of “*Ca.* P. asteris” strains OY-M and AY-WB, “*Ca.* P. australiense” and “*Ca.* P. mali” according to functional categories and sequence homology.

## Figures and Tables

**Table 1:** Overview of the four complete phytoplasma genomes and *A. laidlawii*.

Strain	‘*Candidatus* Phytoplasma' species				*Acholeplasma*
	asteris	asteris	australiense	mali	*laidlawii*
	OY-M	AY-WB	Rp-A	AT	PG-8A
Chromosome organisation	Circular	Circular	Circular	Linear	Circular
Chromosome size	853,092	706,569	879,959	601,943	1,496,992
G + C content (%)	27.76	26.89	27.42	21.39	31.93
G + C % of protein-coding genes^1^	29.09	28.54	28.72	22.58	32.23
Protein-coding genes^1,2^	752	776	684 (155)	481 (16)	1,380 (11)
Protein coding (%)^1^	73.1	73.7	64.1	76.3	90.7
Average ORF size^1^	829	776	825	955	984
Protein-coding genes/kb^1^	0.881	0.949	0.777	0.799	0.921
rRNA operons	2	2	2	2	2
tRNAs	32	31	35	32	34
Accession no.	AP006628.2	CP000061.1	AM422018.1	CU469464.1	CP000896.1
Plasmid-like elements	2	4	1	0	—

^1^Without pseudo genes; ^2^number of annotated pseudo genes is given in brackets.

**Table 2:** Consensus of the DnaA-box motives.

Species/group	DnaA-box	Reference
*Escherichia coli*	5′-TTAT(C/A)CA(C/A)A-3′	[71]
*Mollicutes*	5′-TT(T/A)TC(C/A)ACA-3′	[70]
*A. laidlawii*	5′-TT(T/A)T(C/T)(C/A)ACA-3′	(CP000896)
Candidate motif proposed for phytoplasmas	5′-TTA**GG**AACA-3′	(this study)

**Table 3:** Localization of the rRNA operons.

Product	OY-M	AY-WB	‘*Ca*. P. australiense'	‘*Ca*. P. mali'
16S rRNA	279401–280921	271740–273260	Complement (682149–683667)	264151–265657
23S rRNA	281174–284036	273513–276376	Complement (679085–681932)	265879–268741
5S rRNA	284074–284181	276414–276521^2^	Complement (678945–679050)	268777–268888
16S rRNA	Complement (555991–557511)	Complement (496364–497884)^1^	Complement (863584–865102)	450392–451898
23S rRNA	Complement (552876–555738)	Complement (493248–496111)^1^	Complement (860519–863366)	452120–454982
5S rRNA	Complement (552731–552838)	Complement (493103–493210)^2^	Complement (860380–860484)	455018–455129

rRNA: ribosomal RNA.

^1^Gene was assigned to the opposite strand in contrast to CP000061.

^2^5S-rRNA gene was not annotated in CP000061.
