# Supplementary material for: Current View on Phytoplasma Genomes and Encoded Metabolism
Source: ScientificWorldJournal. 2011 Dec 5;2012:185942. doi: 10.1100/2012/185942 (PMC3322544; doi:10.1100/2012/185942)

Supplementary material- tables and figures

Abbreviations:
'*Candidatus* Phytoplasma species' were abbreviated as '*Ca*. P. asteris' strain OY-M: OY-M; '*Ca*. P. asteris' strain AY-WB: AY-WB; '*Ca*. P. australiense: Paus and '*Ca*. P. mali': Pmal. Locus tags (e.g. PAM_) are provided in brackets.

Supplementary Table 1 (ST1). Retron-related functions.

Gene	Description	OY-M (PAM_)	AYWB (AYWB_)	Paus (PAa_0)	Pmal (ATP_00)
	retron-type reverse transcriptase	342	-	471	-

Supplementary Table 2 (ST2). Genes involved in DNA replication.

Gene	Description	OY-M (PAM_)	AYWB (AYWB_)	Paus (PAa_0)	Pmal (ATP_00)
dnaA	ATPase involved in DNA replication initiation	001	001	0001	193
dnaB1	Replicative DNA helicase	013	007	148	176
dnaB2	Replicative DNA helicase	673	068	822	070
dnaB-like	Replicative DNA helicase	074<br>388<br>550<br>523<br>570<br>645<br>686	179<br>220<br>284<br>617	037<br>073<br>094<br>361	420
dnaC	ATP-binding loader protein for DnaB	672	069	821	069
dnaE	DNA polymerase III, alpha subunit	591	129	535	697
polC	DNA polymerase III, alpha subunit	465	313	541	081<br>422
holA	DNA polymerase III, delta subunit	016	010	145	254
holB	DNA polymerase III, delta prime subunit	231	491	168	374
dnaX	DNA polymerase III, tau subunit	617	117	623	043
dnaN	DNA polymerase sliding camp subunit	002	002	002	194
dnaG	DNA primase	524<br>549<br>569<br>572<br>627<br>644<br>685	108<br>178<br>219<br>285<br>616	614<br>360	305<br>415
dnaD	DNA replication protein BLAST	609	144	630	051
priA	Primosomal protein, replication factor Y	154	565	123	317
ssb	Single-stranded DNA binding protein	010<br>041<br>319	004<br>150<br>193	150<br>814	179<br>398

		324	232		
		359	271		
		364	378		
		396			
		410			
		512			
		535			
		564			
		655			
		699			
		726			
		759			
rnhC	Ribonuclease HIII	631	103	707	130
lig	NAD-dependent DNA ligase	438	321	770	275
gyrA	DNA gyrase subunit A (topoisomerase II)	499	254	214	196
gyrB	DNA gyrase subunit B (topoisomerase II)	498	255	213	195
pmbA	DNA gyrase modulator	764	332	702	324
nrdA	Ribonucleotide reductase, Coenzyme B12-dependent	054	025	056	187
nrdF	Ribonucleotide/diphosphate reductase, subunit beta	055	026	112	186
	putative telomere resolvase	-	-	-	103

Supplementary Table 3 (ST3). Proteins related to chromosome segregation.

**Gene**	**Description**	**OY-M** (PAM_)	**AYWB** (AYWB_)	**Paus** (PAa_0)	**Pmal** (ATP_00)
spoVG	Regulatory protein SpoVG family	732	649	271	227
smc	Chromosome segregation ATPase	693<br>348<br>037<br>516<br>380	277	-	-

Supplementary Table 4 (ST4). Recombination-related functions.

**Gene**	**Description**	**OY-M** (PAM_)	**AYWB** (AYWB_)	**Paus** (PAa_0)	**Pmal** (ATP_00)
ruvA	Holliday junction resolvasome DNA-binding subunit	-	-	-	168
ruvB	Holliday junction resolvasome helicase subunit	-	-	-	167
ruvX	Holliday junction resolvase-like protein	304	418	508	247
recU	Holliday junction-specific endonuclease	-	-	-	209
recA	RecA/RadA recombinase	-	-	-	135
recG	ATP-dependent RecG-like helicase	-	-	-	054
recO	Recombinational DNA repair protein O (RecF pathway)	-	-	-	303
recR	Recombinational DNA repair protein R	-	-	-	045

	(RecF pathway)				
rmuC	RmuC-family protein	270	450	642	-
mgs1	Recombination factor protein RarA	-	288	-	-

Supplementary Table 5 (ST5). DNA repair and degradation.

Gene	Description	OY-M (PAM_)	AYWB (AYWB_)	Paus (PAa_0)	Pmal (ATP_00)
uvrA	Exconuclease ATPase subunit	450	336	699	-
uvrB	Helicase subunit of the DNA excision repair complex	763	399	453	-
uvrC	Helicase subunit of the DNA excision repair complex	433	398	452	-
uvrD	Superfamily I DNA and RNA helicases	315<br>355<br>413<br>505<br>527<br>559<br>621	085<br>115	693	434
mutM	Formamidopyrimidine-DNA glycolase (syn. nei, fbg)	674	067	823	071
mutT	NTP pyrophosphohydrolases including oxidative damage repair enzymes	476	302	776	108
polA	DNA polymerase I - 3'-5' exonuclease and polymerase domain	675<br>676	066	824	072
xseA	Exonuclease VII, large subunit	-	-	-	455
tatD	Mg-dependent DNase	605	140	690	152
srmB	Superfamily II DNA and RNA helicases	453<br>714	054<br>318	155<br>449	028<br>115<br>470
	Putative restriction endonuclease	777	-	-	-
hsdM	Type I restriction-modification system methyltransferase subunit	619	-	-	220
hsdR	Restriction enzymes type I helicase subunits and related helicases	618	-	-	-
ung	Uracil-DNA glycolase	163	556	521	230
nfo	Endonuclease IV	454	317	448	027<br>471

Supplementary Table 6 (ST6). Genes involved in modification and structure.

Gene	Description	OY-M (PAM_)	AYWB (AYWB_)	Paus (PAa_0)	Pmal (ATP_00)
topA	Topoisomerase IA	279	442	634	075
gyrA	DNA gyrase, alpha subunit	499	254	214	196
gyrB	DNA gyrase, beta subunit	498	255	213	195

himA	Nucleotid DNA-binding protein, similar to integration host factor (IHF) and HU	043<br>063<br>242<br>317<br>325<br>357<br>365<br>394<br>402<br>513<br>534<br>563<br>654<br>698<br>758	192<br>210<br>230<br>272<br>377<br>479	042<br>070<br>198<br>230<br>321<br>330<br>376<br>397<br>422<br>432<br>683<br>804	119
	N6 adenine-specific DNA methyltransferase	042<br>299<br>395<br>409<br>565	379<br>424	004<br>043<br>069<br>199<br>231<br>250<br>320<br>327<br>377<br>421<br>433<br>438<br>503<br>739<br>778<br>803	026<br>243<br>262<br>472
-	C-5 cytosine-specific DNA methylase	004	-	-	-
	Putative methyltransferase			291<br>655<br>722<br>789	

Supplementary Table 7 (ST7). RNA polymerase and sigma factors.

Gene	Description	OY-M (PAM_)	AYWB (AYWB_)	Paus (PAa_0)	Pmal (ATP_00)
rpoA	DNA-directed RNA polymerase (α subunit)	227	495	560	367
rpoB	Bifunctional DNA-directed RNA polymerase, β and β' chain	260	461	665	390
rpoC	Bifunctional DNA-directed RNA polymerase, β and β' chain	261	460	664	391
rpoZ	DNA-directed RNA polymerase ω chain	736	654	106	448
rpoE	DNA-directed RNA polymerase, delta subunit	614	119	625	046

rpoD	DNA-dependent RNA polymerase sigma subunits (σ70/32)	628<br>408<br>702<br>760	107<br>194<br>233<br>359<br>380	613<br>258	306<br>397
	RNA polymerase sigma factor, fragment			395	
	RNA polymerase sigma factor			127	
fliA/ rpoF	DNA-directed RNA polymerase specialized sigma subunit	040<br>320<br>323<br>360<br>363<br>511<br>536<br>656<br>700	194<br>206<br>354	258<br>316<br>409<br>723<br>790<br>815	397

Supplementary Table 8 (ST8). Factors affecting the RNA polymerase.

**Gene**	**Description**	**OY-M** (PAM_)	**AYWB** (AYWB_)	**Paus** (PAa_0)	**Pmal** (ATP_00)
greA	Transcription elongation factor	302	420	506	246
nusA	N utilization substance protein A	148	572	606	313
nusB	N utilization substance protein B, transcription termination factor	443	327	835	469<br>029
nusG	Transcription antitermination protein	225	466	670	385
spoT	Guanosine-3',5'-bis(diphosphate) 3'-pyrophosphohydrolase	126	596	767	181

Supplementary Table 9 (ST9). Transcriptional regulators.

**Gene**	**Description**	**OY-M** (PAM_)	**AYWB** (AYWB_)	**Paus** (PAa_0)	**Pmal** (ATP_00)
pepA	Putative aminopeptidase (DNA-binding transcriptional repressor)	-	-	129	431
hrcA	Negative regulator of class I heat shock protein	706	059	557	204
	Putative cold shock protein (transcriptional regulator)	671	070	820	-
	Putative cold shock protein (transcriptional regulator)	-	-	-	295

Supplementary Table 10 (ST10). tRNA synthetases.

Gene	Description	OY-M (PAM_)	AYWB (AYWB_)	Paus (PAa_0)	Pmal (ATP_00)
pheT	Phenylalanyl-tRNA synthetase β-chain	598	134	531	079
pheS	Phenylalanyl-tRNA synthetase α-chain	596	133	532	080
thrS	Threonyl-tRNA synthetase	745	663	018	165
proS	Prolyl-tRNA synthetase	246	475	678	435
alaS	Alanyl-tRNA synthetase	305	416	509	248
hisS	Histidyl-tRNA synthetase	127	594	485	270
glyS	Glycyl-tRNA synthetase β-chain	-	-	-	-
glyQ	Glycyl-tRNA synthetase α-chain	626	109	615	304
argS	Arginyl-tRNA synthetase	455	316	540	436
glnS	Glutaminyl-tRNA synthetase	620	116	694	201
aspS	Aspartyl-tRNA synthetase	128	593	486	271
leuS	Leucyl-tRNA synthetase	738	656	104	446
metG	Methionyl-tRNA synthetase	233	489	128	376
ileS	Isoleucyl-tRNA synthetase	184	535	346	252
cysS	Cysteinyl-tRNA synthetase	141	579	499	335
trpS	Tryptophanyl-tRNA synthetase	466	312	542	092
lysU	Lysyl-tRNA synthetase (class II)	589	128	536	096
gltX	Glutamyl-tRNA synthetase	140	580	498	334
serS	Seryl-tRNA synthetase	083	632	596	132
valS	Valyl-tRNA synthetase	018	012	142	338
tyrS	Tyrosyl-tRNA synthetase, class Ib	469	309	546	114
asnC	asparaginyl-tRNA synthetase	754	671	026	158

Supplementary Table 11 (ST11). tRNAs predicted by tRNAScan-SE in the phytoplasma genomes (differences highlighted in bold letters).

Gene	Amino acid	OY-M	AYWB	Paus	Pmal
tRNA-Ala	Alanine	1	1	1	1
tRNA-Arg	Arginine	2	2	2	2
tRNA-Asn	Asparagine	1	1	1	1
tRNA-Asp	Aspartic acid	1	1	1	1
tRNA-Cys	Cysteine	1	1	1	1
tRNA-Gln	Glutamine	1	1	**2**	1
tRNA-Glu	Glutamic acid	1	1	1	1
tRNA-Gly	Glycine	2	2	2	2
tRNA-His	Histidine	1	1	**2**	1
tRNA-Ile	Isoleucine	2[*1]	2	2	2
tRNA-Leu	Leucine	4	4	**5**	4
tRNA-Lys	Lysine	1	1	1	1
tRNA-Met	Methionine	3	3	3	3
tRNA-Phe	Phenylalanine	1	1	1	1
tRNA-Pro	Proline	1	1	1	1
tRNA-Ser	Serine	3	**3**[*2]	3	3
tRNA-Thr	Threonine	2	2	2	2
tRNA-Trp	Tryptophan	1	1	1	1
tRNA-Tyr	Tyrosine	1	1	1	1

tRNA-Val	Valine	2	2	**1**	2
tRNA-OTHER		/	/	1[*3]	/

[*1] One additional tRNA was predicted at position 281032..281108 (this study)
[*2] One additional tRNA was predicted at position 277264..277354 (this study)
[*3] This tRNA is predicted as Pseudo-tRNA.

Supplementary Table 12 (ST12). Factors affecting the RNA polymerase.

Gene	Description	OY-M (PAM_)	AYWB (AYWB_)	Paus (PAa_0)	Pmal (ATP_00)
trmE	tRNA modification GTPase	747	665	020	164
trmD	tRNA (Guanine-N(1)-)-methyltransferase	471	307	548	439
	tRNA/rRNA methyltransferase protein	672*	468	672	-
spoU	tRNA/rRNA methyltransferase (SpoU)	144	576	-	212
miaA	tRNA delta(2)-isopentenylpyrophosphate transferase	275	446	638	125
truA	Pseudouridylate synthase A	229	493	170	372
truB	tRNA pseudouridine 55 synthase	237	484	783	326
	Probable pseudouridine synthase	238	483	782	123
rluA	pseudouridylate synthase	131	590	489	274
rimM	16S rRNA processing protein	-	-	-	438
ksgA	Dimethyladenosine transferase	731	648	272	226
	Probable Dimethyladenosine transferase	306	415	-	279
Pth	peptidyl-tRNA hydrolase	152	567	610	223
mnmA	tRNA-specific 2-thiouridylase	125	597	766	182

* original annotation differs from this assignment

Supplementary Table 13 (ST13). Translation factors.

Gene	Description	OY-M (PAM_)	AYWB (AYWB_)	Paus (PAa_0)	Pmal (ATP_00)
infA	Translation initiation factor IF-1	223	499	564	363
infB	Translation initiation factor IF-2	150	570	608	315
infC	Translation initiation factor IF-3	744	662	095	441
tufB	Elongation factor Tu	265	456	660	395
tsf	Elongation factor Ts (EF-Ts)	166	553	518	233
lepA	GTP-binding protein LepA (elongation factor)	445	330	222	289
fusA	Elongation factor G 1 (EF-G 1)	264	457	661	394
efp	Translation elongation factor P (EF-P)	276	445	637	124
prfA	Peptide chain release factor 1 (RF-1)	014	008	147	175
prfB	Peptide chain release factor 2 (RF-2)	473	305	550	99
frr	Ribosome recycling factor	168	551	516	235
sua5	Putative translation factor (SUA5)	015	009	146	174
rbfA	Ribosome-binding factor A	151	569	609	316

Supplementary Table 14 (ST14). Ribosomal proteins.

Gene	Description	OY-M (PAM_)	AYWB (AYWB_)	Paus (PAa_0)	Pmal (ATP_00)
rplA	50S ribosomal protein L1	257	464	668	387
rplB	50S ribosomal protein L2	203	519	584	344
rplC	50S ribosomal protein L3	200	522	587	341
rplD	50S ribosomal protein L4	201	521	586	342
rplE	50S ribosomal protein L5	212	510	575	353
rplF	50S ribosomal protein L6	215	507	572	356
rplI	50S ribosomal protein L9	012	006	149	177
rplJ	50S ribosomal protein L10	258	463	667	388
rplK	50S ribosomal protein L11	256	465	669	386
rplL	50S ribosomal protein L7/L12	259	462	666	389
rplM	50S ribosomal protein L13	138	582	496	332
rplN	50S ribosomal protein L14	210	512	577	351
rplO	50S ribosomal protein L15	219	503	568	360
rplP	50S ribosomal protein L16	207	515	580	348
rplQ	50S ribosomal protein L17	228	494	559	368
rplR	50S ribosomal protein L18	216	506	571	357
rplS	50S ribosomal protein L19	470	308	547	440
rplT	50S ribosomal protein L20	742	660	097	443
rplU	50S ribosomal protein L21	086	629	594	284
rplV	50S ribosomal protein L22	205	517	582	346
rplW	50S ribosomal protein L23	202	520	585	343
rplX	50S ribosomal protein L24	211	511	576	352
rpmA	50S ribosomal protein L27	088	627	592	282
rpmB	50S ribosomal protein L28	106	603	715	278
rpmC	50S ribosomal protein L29	208	514	579	349
rpmD	50S ribosomal protein L30	218	504	569	359
rpmE	50S ribosomal protein L31	105	604	714	141
rpmF	50S ribosomal protein L32	198	525	589	308
rpmG	50S ribosomal protein L33	244<br>253	476	680	383
rpmH	50S ribosomal protein L34	247	474	677	378
rpmI	50S ribosomal protein L35	743	661	096	442
rpmJ	50S ribosomal protein L36	224	498	563	364
rpsB	30S ribosomal protein S2	165	554	519	232
rpsC	30S ribosomal protein S3	206	516	581	347
rpsD	30S ribosomal protein S4	586	241	088	310
rpsE	30S ribosomal protein S5	217	505	570	358
rpsF	30S ribosomal protein S6	009	003	152	180
rpsG	30S ribosomal protein S7	263	458	662	393
rpsH	30S ribosomal protein S8	214	508	573	355
rpsI	30S ribosomal protein S9	139	581	497	333
rpsJ	30S ribosomal protein S10	199	524	588	340
rpsK	30S ribosomal protein S11	226	496	561	366
rpsL	30S ribosomal protein S12	262	459	663	392
rpsM	30S ribosomal protein S13	225	497	562	365
rpsN	30S ribosomal protein S14	213	509	574	354
rpsO	30S ribosomal protein S15	136	585	494	269
rpsP	30S ribosomal protein S16	472	306	549	437

rpsQ	30S ribosomal protein S17	209	513	578	350
rpsR	30S ribosomal protein S18	011	005	150	178
rpsS	30S ribosomal protein S19	204	518	583	345
rpsT	30S ribosomal protein S20	081	633	599	133
rpsU	30S ribosomal protein S21	623	112	618	300

Supplementary Table 15 (ST15). Modifying and accessory factors.

Gene	Description	OY-M (PAM_)	AYWB (AYWB_)	Paus (PAa_0)	Pmal (ATP_00)
era	GTP-binding protein Era (cell cycle/ribosome assembly)	625	110	616	302
engA	GTP-binding protein EngA (ribosome assembly)	240	481	780	121
engB	ribosome biogenesis GTP-binding protein	017	011	143	102
engC	GTPase EngC	182*	537	348	078
engD	GTP-dependent nucleic acid-binding protein EngD	183	536	347	251
obgE	GTPase ObgE	089	626	591	281
	ribosome biogenesis GTP-binding protein YlqF/GTPase	278*	443	635	076*

* original annotation differs from this assignment; this assignment is based on sequence homology and Pfam.

Supplementary Table 16 (ST16). Modifying and accessory factors.

Gene	Description	OY-M (PAM_)	AYWB (AYWB_)	Paus (PAa_0)	Pmal (ATP_00)
YkqC	RNase J1/J2	633*	101*	011*	211*

* original annotation differs from this assignment

Supplementary Table 17 (ST17). Heat shock proteins and chaperons.

Gene	Description	OY-M (PAM_)	AYWB (AYWB_)	Paus (PAa_0)	Pmal (ATP_00)
dnaJ	Chaperone DnaJ	703	062	554	207
dnaK	Chaperone DnaK	704	061	555	206
grpE	Chaperone GrpE	705	060	556	205
groeL	Chaperonin 60kD subunit	121	600	761	185
groeS	Chaperonin 10kD subunit	120	601	760	184
	Trigger factor (TF)	449	334	700	297
lon	ATP-dependent Lon protease	448	333	701	298
ibpA	Molecular chaperone	028	017	717	-

Supplementary Table 18 (ST18). Other proteins involved in translation.

Gene	Description	OY-M (PAM_)	AYWB (AYWB_)	Paus (PAa_0)	Pmal (ATP_00)
map	Methionine aminopeptidase	222	500	565	362
rnpA	Ribonuclease P protein component	248	473	676	379
pnp	Polyribonucleotide nucleotidyltransferase	251	470	674	381
pcnB	Poly(A)polymerase	102	607	711	238
smpB	SsrA-binding protein	146	574	603	328

Supplementary Table 19 (ST19). Cell envelelope reated- proteins.

Gene	Description	OY-M (PAM_)	AYWB (AYWB_)	Paus (PAa_0)	Pmal (ATP_00)
mraW	Methyltransferase involved in cell envelope biogenesis	735	652	107	449

Supplementary Table 20 (ST20). Proteins involved in lipopolysaccharide synthesis.

Gene	Description	OY-M (PAM_)	AYWB (AYWB_)	Paus (PAa_0)	Pmal (ATP_00)
rfaG	Glycosyltransferase involved in LPS biosynthesis	008	-	-	083

Supplementary Table 21 (ST21). Proteins involved in cell division.

Gene	Description	OY-M (PAM_)	AYWB (AYWB_)	Paus (PAa_0)	Pmal (ATP_00)
ftsH	Cell division protein FtsH homolog	591*	130	488*	273*
ftsY	Cell division protein FtsY homolog	064	679	827	074
gidB	Methyltransferase, glucose inhibited division protein B	267	454	645	-
gidA	Glucose inhibited division protein A	268	453	644	137

* original annotation differs from this assignment

Supplementary Table 22 (ST22). Immunodominant membrane proteins.

Gene	Description	OY-M (PAM_)	AYWB (AYWB_)	Paus (PAa_0)	Pmal (ATP_00)
amp	antigenic membrane protein/ immunodominant membrane protein	122	599	762	-
imp	immunodominant membrane protein	-	-	-	050
	ArsC family/Spx protein				

Supplementary Table 23 (ST23). Predicted integral membrane proteins caring a single transmembrane helix (TM) at least.

OY-M (PAM_)	TM(s)	AYWB (AYWB_)	TM(s)	Paus (PAa_0)	TM(s)	Pmal (ATP_00)	TM(s)
005	6	006	2	015	1	002	5
006	6	013	5	016	1	003	9
012	2	015	4	017	1	004	8
019	4	018	10	019	2	007	4
021	1	019	1	023	6	009	7
022	3	023	1	024	6	011*	1
027	2	024	2	027	1	012	2
029	1	027	3	047	1	013	4
030	10	029	6	048	1	014	3
031	1	042	1	065	2	015	6
046	2	052	13	080	2	017	2
048	1	053	6	081	1	018	6
057	7	055	1	083	2	019	12
058	3	063	1	086	1	020	1
061	2	073	1	089	10	021	1
065	1	080	1	090	3	022	1
066	2	081	2	092	2	023	2
068	1	082	1	098	6	032	6
077	3	083	7	109	3	033	1
078	6	084	1	110	12	034	2
082	1	091	1	111	1	035	1
092	1	092	2	112	1	036	1
094	19	093	6	113	2	038	1
095	1	094	6	114	6	039	1
096	4	096	1	115	5	040	1
102	1	105	1	116	1	041	1
107	1	120	6	119	1	047	7
109	1	121	1	120	7	048	1
111	1	124	2	125	2	050	1
114	2	125	6	130	2	056	1
119	6	132	3	132	1	058	1
126	1	135	4	133	6	061	3
130	2	145	1	134	6	066	6
134	5	146	1	136	2	067	6
153	4	148	1	137	2	068	1
156	1	149	1	139	4	076	1
157	1	152	1	141	5	077	1

158	7	153	1	149	2	082	6
162	3	155	1	153	2	084	6
169	9	157	1	154	1	085	6
173	1	158	1	156	1	086	1
176	6	159	1	157	1	087	1
177	4	160	1	158	1	088	4
180	9	161	1	160	1	098	3
186	10	162	7	162	1	101	4
189	6	166	2	163	1	103	1
190	6	167	1	165	10	104	13
191	1	173	1	168	1	107	12
196	1	177	1	171	11	110	6
220	10	183	2	173	9	111	6
232	1	185	3	177	5	127	1
235	2	186	1	178	5	128	1
241	1	187	1	180	1	129	2
243	5	188	1	186	2	134	6
249	7	189	1	192	1	136	1
252	11	190	1	202	1	138	2
254	3	202	1	203	1	143	2
258	1	211	1	204	1	145	1
269	2	212	1	205	2	146	1
270	1	214	1	218	1	149	1
272	1	218	1	219	1	150	2
277	1	224	1	220	2	151	13
280	12	225	1	221	1	160	6
282	12	228	2	223	6	161	6
286	8	229	1	234	1	169	1
288	1	236	1	235	1	173	5
289	1	237	1	238	2	177	2
297	1	242	10	241	1	183	1
298	1	243	3	242	2	188	1
306	7	256	2	243	1	191	5
307	2	257	2	244	1	192	1
308	6	261	2	246	1	203	7
311	2	262	5	251	1	221	10
312	1	263	1	266	6	222	8
313	7	265	5	267	5	228	8
314	1	269	1	268	1	229	2
316	1	274	1	269	2	236	9
326	1	275	1	270	8	241	1
327	2	276	2	312	1	242	1
329	1	279	2	313	1	253	5
347	1	292	1	314	1	259	1
349	2	294	1	318	7	265	2
351	2	297	1	329	1	267	1
352	1	300	1	333	1	272	2
353	6	303	1	336	1	273	2
354	1	314	6	337	1	276	3
366	1	315	6	339	1	279	7
367	2	316	1	345	10	280	5
369	1	319	2	380	1	291	6
378	1	320	6	381	1	292	6
379	2	329	1	382	1	293	2
382	1	342	1	383	2	299	2
383	2	345	2	385	2	309	5

392	1	351	1	389	1	311	10
403	1	353	2	390	1	312	3
404	1	357	1	393	1	318	7
405	2	358	4	398	1	320	1
412	1	366	1	407	7	322	4
414	1	368	1	416	1	327	1
415	4	369	1	417	1	330	5
416	1	371	1	418	1	331	2
417	2	373	2	436	1	361	9
418	2	376	1	438	1	371	9
419	1	385	2	440	1	375	1
755	1	392	1	446	2	379	1
756	1	404	3	450	6	380	7
761	2	405	2	457	2	382	1
436	5	408	3	467	1	384	3
437	1	409	1	481	1	388	1
444	1	413	6	483	9	396	1
446	2	414	2	484	9	400	1
457	6	415	7	487	1	401	3
458	2	425	1	488	2	404	1
460	3	426	1	492	6	405	1
461	1	432	2	501	1	406	1
462	2	433	1	502	1	412	2
475	1	435	11	515	9	413	1
477	1	439	12	522	2	416	1
479	1	441	12	526	1	427	7
483	2	444	1	530	4	429	4
485	1	448	1	533	3	433	5
487	5	450	1	538	5	444	6
489	1	452	2	539	2	453	1
490	5	455	7	541	1	456	1
494	1	463	1	543	5	459	1
495	1	467	3	552	1	460	1
507	1	468	1	553	1	463	1
508	6	469	10	558	1	464	2
509	1	471	2	567	10	465	1
510	2	472	7	590	3	466	6
515	2	477	6	597	1	475	2
517	1	479	1	598	1	476	1
528	1	488	2	600	1	477	1
529	3	490	1	604	6	478	1
530	3	529	1	626	7	479	12
531	2	530	6	629	1	480	6
532	1	531	6	633	12	481	2
539	1	533	11	635	1	483	6
541	1	534	6	636	1	484	3
555	2	539	9	640	1	485	4
556	1	543	5	641	1	486	2
557	7	544	6	642	1	489	7
558	1	550	9	643	2	491	4
561	1	557	2	648	1	494	8
562	1	559	1	649	1	495	9
575	1	561	8	671	3	496	5
580	1	562	1	673	9		
584	3	566	4	681	6		
585	10	568	1	685	1		

587	3	583	2	692	2
588	6	586	1	695	3
595	3	587	5	696	4
599	5	591	2	697	1
604	2	599	2	704	1
610	1	602	6	706	1
613	7	615	1	707	1
638	1	619	4	709	2
639	4	620	2	729	1
649	1	621	9	742	1
650	1	622	9	749	2
652	2	624	1	755	1
657	1	635	6	756	1
658	1	636	1	757	6
660	6	650	8	758	6
661	6	651	10	768	13
662	2	659	6	772	1
663	1	667	1	774	2
670	1	668	6	779	10
680	4	669	6	785	1
691	2			792	7
694	2			797	2
696	1			798	3
707	1			799	1
718	6			800	1
719	1			813	7
720	10			816	6
727	1			817	1
733	8			822	1
734	12			829	1
735	1			834	1
741	6			839	1
749	1				
751	6				
753	1				

Supplementary Table 24 (ST24). Predicted integral membrane proteins caring a single transmembrane helix (TM) and a signal peptide at least.

OY-M (PAM_)	TM(s)	AYWB (AYWB_)	TM(s)	Paus (PAa_0)	TM(s)	Pmal (ATP_00)	TM(s)
016	1	023	5	138	1	010	1
028	5	059	5	406	1	117	5
114	1	091	1	460	3	148	1
191	1	122	1	523	1	376	1
383	3	185	5	611	3	488	1
395	1	266	6	675	6		
428	1	295	1	762	1		
502	9	456	5	812	1		
625	1	464	2	818	1		
		497	5	828	1		
		502	1				
		750	5				

Supplementary Table 25 (ST25). Secretion apparatus.

Gene	Description	OY-M (PAM_)	AYWB (AYWB_)	Paus (PAa_0)	Pmal (ATP_00)
secA	Preprotein translocase subunit SecA	474	304	551	100
secE	Preprotein translocase subunit SecE	254	467	671	384
secY	Preprotein translocase subunit SecY	220	502	567	361
yidC	Preprotein translocase subunit yidC	249	472	675	380

Supplementary Table 26 (ST26). Predicted secreted proteins.

OY-M (PAM_)	AYWB (AYWB_)	Paus (PAa_0)	Pmal (ATP_00)
049	022		
069	032		
070	033	022	
125	122	030	
133	127	060	
160	129	064	
161	131	074	006
195	168	105	025
233	169	185	031
271	174	193	037
330	203	217	049
331	240	225	090
332	245	255	120
346	258	263	147
356	259	276	182
370	280	288	189
371	295	310	197
372	339	326	257
393	340	331	258
484	346	335	261
486	367	369	266
514	370	396	268
518	387	402	287
519	402	469	426
540	449	478	447
542	480	491	458
576	489	603	461
577	558	628	462
582	588	708	467
591	595	726	473
611	597	728	492
643	638	748	
653	640	766	
690	645	809	
710	655	837	
737	658		
765			

Supplementary Table 27 (ST27). Signal recognition particle (SRP) pathway associated proteins.

Gene	Description	OY-M (PAM_)	AYWB (AYWB_)	Paus (PAa_0)	Pmal (ATP_00)
ftsY	Cell division protein, SRP docking protein	679	064	827	074
ffh	Signal recognition particle protein	678	065	826	073
yidC	Preprotein translocase subunit YidC	249	472	675	380

Supplementary Table 28 (ST28). Putative complete ABC transporter subunits with assignment.

Gene	Description	OY-M (PAM_)	AYWB (AYWB_)	Paus (PAa_0)	Pmal (ATP_00)
potA	ABC-type spermidine/putrescine transport system, ATPase component	659	095	135	290
potB	ABC-type spermidine/putrescine transport system, permease component I	660	094	134	291
potC	ABC-type spermidine/putrescine transport system, permease component II	661	093	133	292
potD	ABC-type spermidine/putrescine transport system, periplasmatic component	662	092	132	293
znuA	ABC-type Mn/Zn transport system, periplasmatic component	092	624	481	492
znuC	ABC-type Mn/Zn transport system, ATPase component	093	623	482	493
znuB	ABC-type Mn/Zn transport system, permease component	094	622<br>621	483<br>484	494<br>495
znuA2	ABC-type Mn/Zn transport system, periplasmatic component	/	/	/	006
znuC2	ABC-type Mn/Zn transport system, ATPase component	/	/	/	005
znuB2	ABC-type Mn/Zn transport system, permease component	/	/	/	004<br>003
malK	ABC-type sugar transport system (probably encoding maltose/maltodextrin transporter subunit, also similar to glycerol-3-phosphate transporter UgpC subunit), ATPase component	752	670	025	159
malF	ABC-type sugar transport system (probably encoding maltose/maltodextrin transporter subunit, also similar to glycerol-3-phosphate transporter UgpA subunit), permease component I	751	669	024	160
malG	ABC-type sugar transport system(probably encoding maltose/maltodextrin transporter subunit, also similar to glycerol-3-phosphate transporter UgpE subunit), permease component II	750	668	023	161

malE	ABC-type sugar transport system (probably encoding maltose/maltodextrin transporter subunit, also similar to glycerol-3-phosphate transporter UgpB subunit), periplasmatic component	749	667	022	162
dppF	ABC-type dipeptide/oligopeptide transport system, ATPase component I	193*	527	753	065
dppD	ABC-type dipeptide/oligopeptide transport system, ATPase component II	192	528	754	064
dppA	ABC-type dipeptide/oligopeptide transport system, periplasmatic component	191	529	755<br>756	068
dppB	ABC-type dipeptide/oligopeptide transport system, permease component I	190	530	757	067
dppC	ABC-type dipeptide/oligopeptide transport system, permease component II	189	531	758	066
metN	ABC-type methionine transport system, ATPase component	132	589	490	157
metQ	ABC-type methionine transport system, periplasmatic component	133	588	491	192
metI	ABC-type methionine transport system, permease component	134	587*	492	191
artM	ABC-type amino acid transport system (probably arginine transporting), permease component I	490	262	450	/
artI	ABC-type amino acid transport system (probably arginine transporting), periplasmatic component	489	263	267*	/
artP	ABC-type amino acid transport system (probably arginine transporting), ATPase component	488	264	775	/
artQ	ABC-type amino acid transport system (probably arginine transporting), permease component II	487	265	/	/
artM2	ABC-type amino acid transport system (probably arginine transporting), permease component I	077	/	/	/
artI2	ABC-type amino acid transport system (probably arginine transporting), periplasmatic component	076*	/	/	/
artP2	ABC-type amino acid transport system (probably arginine transporting), ATPase component	079	634	/	/
	ABC-type amino acid transport system similar to ArtQ, permease and periplasmatic component	078	635	/	/

* original annotation differs from this assignment

Supplementary Table 29 (ST29). Identified subunits of probably incomplete ABC transporters.

Gene	Description	OY-M (PAM_)	AYWB (AYWB_)	Paus (PAa_0)	Pmal (ATP_00)
dppF2	ABC-type dipeptide/oligopeptide transport system, ATPase component I	026	/	718	/
dppD2	ABC-type dipeptide/oligopeptide transport system, ATPase component II	025	/	/	/
dppB2	ABC-type dipeptide/oligopeptide transport system, permease component I	022	/	/	/
dppC2	ABC-type dipeptide/oligopeptide transport system, permease component II	023[*1]	/	/	/
oppA	ABC-type oligopeptide transport system, periplasmatic component	/	/	/	463
cbiQ	ABC-type cobalt transport system, permease component	180	539	173	371
cbiO	ABC-type cobalt transport system, ATPase component	179[*1]	540<br>541	174<br>175	370<br>369
cbiQ2	ABC-type cobalt transport system, permease component	[*1]	015	139	/
cbiO2	ABC-type cobalt transport system, ATPase component	020	014	140	/
artM	ABC-type amino acid transport system (probably arginine transporting), permease component	494	/	/	/
artI	ABC-type amino acid transport system (probably arginine transporting), periplasmatic component	495	/	/	/
	ABC-type amino acid transport system similar to ArtM, permease and periplasmatic component	456	315	/	/
	ABC-type amino acid transport system, permease component		314[*2]		
	ABC-type amino acid transport system similar to ArtI, permease and periplasmatic component	457	/	/	/
artM	ABC-type amino acid transport system (probably arginine transporting), permease component	639	/	/	/
	ABC-type transport system, periplasmatic component	640[*2]	/	/	/
	ABC-type amino acid transport system, periplasmatic component	641	/	/	/
artM	ABC-type amino acid transport system (probably arginine transporting), permease component	464	/	/	/
	ABC-type amino acid transport system similar to ArtM, permease and periplasmatic component	588	125	/	/
	ABC-type amino acid transport system (periplasmatic component), fragment-pseudo	/	/	013[*2]	/
artM	ABC-type amino acid transport system	/	/	014	/

	(permease component), fragment-pseudo				

*1 indicates that the subunit was identified by additional analysis but is not part of the current submitted annotation in the databases.
*2 * original annotation differs from this assignment

Supplementary Table 30 (ST30). Putative thiamin transporter subunit.

Gene	Description	OY-M (PAM_)	AYWB (AYWB_)	Paus (PAa_0)	Pmal (ATP_00)
-	Putative thiamine transporter protein,Thia_YuaJ family	176*	544*	178*	309*

* original annotation differs from this assignment

Supplementary Table 31 (ST31). Symporters.

Gene	Description	OY-M (PAM_)	AYWB (AYWB_)	Paus (PAa_0)	Pmal (ATP_00)
mleP	Malate (citrate)/Na+ symporter	720<br>286	052<br>435	768	104<br>151<br>019<br>479

Supplementary Table 32 (ST32). Multidrug efflux pump proteins.

Gene	Description	OY-M (PAM_)	AYWB (AYWB_)	Paus (PAa_0)	Pmal (ATP_00)
mdlB	multidrug efflux pump, ATP-binding and permease protein	059	028	114	111
mdlA	multidrug efflux pump, ATP-binding and permease protein	*	029	115	110
norM	Na+-driven multidrug efflux pump	280<br>282<br>734	441<br>439<br>651	633<br>165<br>110<br>171	107

* indicates that the subunit was identified by additional analysis; original annotation differs from this assignment

Supplementary Table 33 (ST33). P-type ATPases.

Gene	Description	OY-M (PAM_)	AYWB (AYWB_)	Paus (PAa_0)	Pmal (ATP_00)
mgtA	P-type ATPase exporting cations (probably Mg)	585	242	089	311
	P-type ATPase exporting cations (probably calcium)	186	533	345	221
	P-type ATPase exporting cations	030<br>252	018<br>469	779<br>673	222
	P-type ATPase exporting cations (probably cadmium)	733	650	270	228

Supplementary Table 34 (ST34). Large-conductance mechanosensitive channel proteins.

Gene	Description	OY-M (PAM_)	AYWB (AYWB_)	Paus (PAa_0)	Pmal (ATP_00)
mscL	large-conductance mechanosensitive channel	058	027	113	331

Supplementary Table 35 (ST35). Glycolysis and related genes.

Gene	Description	OY-M (PAM_)	AYWB (AYWB_)	Paus (PAa_0)	Pmal (ATP_00)
-	Had superfamily family phosphatase, subfamily IIIa	301	421	505	245
Cof	Had superfamily hydrolase, Subfamily IIb (sucrose-phosphate phosphatase)	171<br>593<br>634	548<br>100	010<br>513<br>534	106
pgi	glucose-6-phosphate isomerase	283	438	166	377
pfkA	6-phosphofructokinase	281	440	164	105
fba	Fructose-bisphosphate aldolase	172	547	512	109
tpiA	triosephosphate isomerase	170	549	514	166
gapA	glyceraldehyde-3-phosphate dehydrogenase	175	545	510	-
pgk	phosphoglycerate kinase	174	546	511	-
pgm (gpmI)	Phosphoglyceromutase	285	436	060	-
eno	phosphopyruvate hydratase	284	437	059	-
pykF	pyruvate kinase	287	434	061	-
dhaK	kinase related to dihydroxyacetone kinase	665	090	819	277
gtfA	Sucrose phosphorylase (Glucosyltransferase-A)	723	-	221	-

Supplementary Table 36 (ST36). Deduced proteins involved in the formation of acetate from malate.

Gene	Description	OY-M (PAM_)	AYWB (AYWB_)	Paus (PAa_0)	Pmal (ATP_00)
mleP	Malate (citrate)/Na+ symporter	720<br>286	052<br>435	768	104<br>151<br>019<br>479
sfcA (maeA)	NAD+-dependent malic enzyme	721*	051	769	450*
acoB (pdhB)	thiamine pyrophosphate-dependent dehydrogenase, E1 component beta subunit	601	137	687	155
acoA (pdhA)	thiamine pyrophosphate-dependent dehydrogenase, E1 component alpha subunit (pyruvate dehydrogenase)	600	136	686	156
aceF (pdhC)	branched-chain alpha-keto acid dehydrogenase subunit E2 (lipoate acetyltransferase)	602	138	688	154
Lpd (pdhD)	Dihydrolipoamide dehydrogenase	603	139	689	153
coaE	coenzyme A kinase*	674	067	823	071
hcaD	NADH oxidase H_2O_2-forming	441	324	837*	288
pduL-like	CoA-phosphotransacetylase, similar to propanediol utilisation protein PduL	729*	646*	274*	224*
ackA	Acetate kinase	159	560	121	319
eda	2-dehydro-3-deoxyphosphogluconate aldolase/4-hydroxy-2-oxoglutarate aldolase	-	-	-	116
acoB (pdhB)	thiamine pyrophosphate-dependent dehydrogenase, E1 component beta subunit	601	137	687	155
acoA (pdhA)	thiamine pyrophosphate-dependent dehydrogenase, E1 component alpha subunit (pyruvate dehydrogenase)	600	136	686	156
aceF (pdhC)	branched-chain alpha-keto acid dehydrogenase subunit E2 (lipoate acetyltransferase)	602	138	688	154
Lpd (pdhD)	Dihydrolipoamide dehydrogenase	603	139	689	153
coaE	coenzyme A kinase*	674	067	823	071
hcaD	NADH oxidase H_2O_2-forming	441	324	837*	288
pduL-like	CoA-phosphotransacetylase, similar to propanediol utilisation protein PduL	729*	646*	274*	224*
ackA	Acetate kinase	159	560	121	319

* original annotation differs from this assignment

Supplementary Table 37 (ST37). NAD synthesis.

Gene	Description	OY-M (PAM_)	AYWB (AYWB_)	Paus (PAa_0)	Pmal (ATP_00)
nadE/ qns	Glutamine-dependent NAD+ synthetase	124	598	763	183

Supplementary Table 38 (ST38). Superoxide dismutase.

Gene	Description	OY-M (PAM_)	AYWB (AYWB_)	Paus (PAa_0)	Pmal (ATP_00)
sodA	superoxide dismutase	442	326	836	468<br>030

Supplementary Table 39 (ST39). Lipoyl-protein ligases.

Gene	Description	OY-M (PAM_)	AYWB (AYWB_)	Paus (PAa_0)	Pmal (ATP_00)
lplA	lipoate-protein ligase A	309	412	537	208

Supplementary Table 40 (ST40). Nucleotide metabolism.

Gene	Description	OY-M (PAM_)	AYWB (AYWB_)	Paus (PAa_0)	Pmal (ATP_00)
adk	adenylate kinase	221	501	566	031<br>467
gmk	guanylate kinase	680<br>737	655	105	447
pnp	Polyribonucleotide nucleotidyltransferase	251	470	674	381
nrdA	Ribonucleotide reductase, Coenzyme B12-dependent	054	025	056	187
nrdF	Ribonucleotide/diphosphate reductase, subunit beta	055	026	112	186
spoT	Guanosine-3',5'-bis(diphosphate) 3'-pyrophosphohydrolase	126	596	767	181
udk	uridine kinase	142*	578	-	-
pyrH	Uridylate kinase	167	552	517	234
pyrG	CTP synthase	611	122	628	049
cmk	cytidylate kinase	239	482	781	122
thyA	thymidylate synthase	293	430	084	320
tdk	thymidine kinase	104	605	713	140
tmk	Thymidylate kinase	230<br>344<br>374<br>521	074<br>181<br>222<br>282<br>492	071<br>169<br>304<br>324<br>365	373<br>403

Gene	Description	OY-M	AYWB	Paus	Pmal
				373<br>425<br>807<br>431<br>735<br>195<br>039<br>227	
dut	DUTPase	164	555	520	231
hit	diadenosine tetraphosphate hydrolase (AppppA hydrolase)	630	104	705	131
-	CMP/dCMP deaminase zinc-binding protein	103	606	712	139
	Putative IMP dehydrogenase/GMP reductase	-	-	048<br>068<br>203<br>381<br>417	-

* original annotation differs from this assignment

Supplementary Table 41 (ST41). Peptidases.

Gene	Description	OY-M (PAM_)	AYWB (AYWB_)	Paus (PAa_0)	Pmal (ATP_00)
pepP	Xaa-Pro aminopeptidase	245	-	679	042
pepV	dipeptidase PepV	290	431	-	-
oepF	oligoendopeptidase F	178	542	176	-

Supplementary Table 42 (ST42). HflB and TldD proteins grouped by BLASTClust.

Gene	Description	OY-M (PAM_)	AYWB (AYWB_)	Paus (PAa_0)	Pmal (ATP_00)
hflB/ ftsH	ATP-dependent zinc metallopeptidase, transmembrane protein, HflB-like enzymes	064<br>117<br>130<br>307<br>316<br>326<br>356<br>366<br>393<br>403<br>480<br>532<br>561<br>658<br>664*<br>695	071<br>096<br>183<br>187<br>213<br>229<br>275<br>409<br>414<br>591	136<br>235<br>488<br>523<br>539<br>799	011<br>034<br>039<br>146<br>273<br>382<br>406<br>464<br>460<br>487

		713<br>727<br>762			
tldD	Zn-dependent protease	447*	331*	703	323

* original annotation differs from this assignment

Supplementary Table 43 (ST43). Amino acid synthesis and modification.

Gene	Description	OY-M (PAM_)	AYWB (AYWB_)	Paus (PAa_0)	Pmal (ATP_00)
metK	S-Adenosylmethionine synthase; methionine adenosyltransferase	-	-	-	296
asnB	Asparagine synthase	594	130	-	-
smtA	SAM-dependent methyltransferase	303	419	507	-
argE	acetylornithine deacetylase	292	-	-	-

Supplementary Table 44 (ST44). Genes involved in lipid synthesis.

Gene	Description	OY-M (PAM_)	AYWB (AYWB_)	Paus (PAa_0)	Pmal (ATP_00)
cdsA	Phosphatidate cytidylyltransferase	169	550	515	236
pgsA	CDP-diacylglycerol-glycerol-3-phosphatidyltransferase	718	053	816	134
plsC	Putative 1-acyl-sn-glycerol-3-phosphate acyltransferase	295	428	086	322
plsX	Fatty acid/phospholipid synthesis protein	607	142	632	053
plsY	acyl phosphate:glycerol-3-phosphate acyltransferase	436*	320*	266*	173*
psD	Phosphatidylserine decarboxylase proenzyme	612	121	627	048
pssA	CDP-diacylglycerol-serine O-phosphatidyltransferase	613	120	626	047
gpsA	glycerol 3-phosphate dehydrogenase	241	480	684	120

*The gene *plsY* is annotated within the four genomes (and *A. laidlawii,* ACL_0547) as hypothetical protein.

Supplementary Table 45 (ST45). Genes involved in lipid synthesis.

Gene	Description	OY-M (PAM_)	AYWB (AYWB_)	Paus (PAa_0)	Pmal (ATP_00)
ribF	Riboflavin kinase + FAD synthetase	451	-	-	-

Supplementary Table 46 (ST46). Genes involved in lipid synthesis.

Gene	Description	OY-M (PAM_)	AYWB (AYWB_)	Paus (PAa_0)	Pmal (ATP_00)
glyA	serine hydroxymethyltransferase	181	538	172	142
folk	7,8-Dihydro-6-hydroxymethylpterin-pyrophosphokinase	580	249*	-	-
folC	Putative bifunctional protein: folylpolyglutamate synthase and dihydrofolate synthase	007	-	-	-
folA	dihydrofolate reductase	294	429	085	321
folP	dihydropteroate synthase	581	246*	-	-

* original annotation differs from this assignment

Supplementary Table 47 (ST47). Thiamine synthesis.

Gene	Description	OY-M (PAM_)	AYWB (AYWB_)	Paus (PAa_0)	Pmal (ATP_00)
thiJ	4-methyl-5(B-hydroxyethyl)-thiazole monophosphate biosynthesis enzyme	137*	584	495*	237
thiI	thiamine biosynthesis protein	143*	577	601	-

* original annotation differs from this assignment

Supplementary Table 48 (ST48). Pyridoxalphosphate.

Gene	Description	OY-M (PAM_)	AYWB (AYWB_)	Paus (PAa_0)	Pmal (ATP_00)
pdxK	pyridoxal/pyridoxine/pyridoxamine kinase	434	-	-	-

Supplementary Table 49 (ST49). Proteins involved in in iron-sulfur cluster biosynthesis.

Gene	Description	OY-M (PAM_)	AYWB (AYWB_)	Paus (PAa_0)	Pmal (ATP_00)
(iscU)	nifU-like protein, involved in Fe-S cluster formation	467<br>100<br>609	609	544<br>456	112
(csdB)	nifS-like protein (selenocysteine lyase)	468	310	545	113

Supplementary Table 50 (ST50). Acyl carrier protein metabolism.

Gene	Description	OY-M (PAM_)	AYWB (AYWB_)	Paus (PAa_0)	Pmal (ATP_00)
acpP	Acyl carrier protein	739	657	099	445
acpS	Holo-(acyl-carrier-protein) synthase	440	323	838	294

Supplementary Table 51 (ST51). Phosphorus metabolic processes.

Gene	Description	OY-M (PAM_)	AYWB (AYWB_)	Paus (PAa_0)	Pmal (ATP_00)
ppa	inorganic pyrophosphatase	145	575	602	329
fba	fructose/tagatose bisphosphate aldolase	172	547	512	109
	exopolyphosphatase-related protein	746	664	019	163
	exopolyphosphatase-related protein	748	666	021	-
hd	superfamily phosphohydrolase	730	647	273	225
	phosphodiesterase	272	448	640	127
	Putative metallophosphoesterase	273*	447*	639	126*
pgpB	membrane-associated phospholipid phosphatase	005	-	-	085
pldB	Putative lysophospholipase	-	-	-	339
pldB	Putative lysophospholipase	-	-	-	396

* original annotation differs from the assignment

Supplementary Table 52 (ST52). Miscellaneous.

Gene	Description	OY-M (PAM_)	AYWB (AYWB_)	Paus (PAa_0)	Pmal (ATP_00)
frvX	endo-1,4-beta-glucanase-like protein	740	658	-	-
trx	Thioredoxin	236	485	784	325
	tengu-su inducer	765	168	-	-
	Zn-dependent hydrolase	637	098	006	210
YjeE	P-loop hydrolase	500	253	215	198
	Uncharacterised protein, DegV family	583	244	091	095
	Uncharacterised protein, DegV family	636	099	007	-
gcp	O-sialoglycoprotein endopeptidase	579	251	093	200
	Conserved hypothetical protein, similar to 3'-5' exoribonuclease YhaM	101	608	710	239
tpm	tetrapyrrole (Corrin/Porphyrin) methylase	232	490	167	375
	Conserved hypothetical protein	584	243	090	312
	glycoprotease family protein	501	252	216	199
	metalloprotein	624*	111	617	301*
	Nitroreductase	435	397	500	286
	Nitroreductase-like protein	-	-	-	287
arsC	Putative arsenate reductase related protein,thioredoxin family [hypoth. protein]	632	-	012	202

* original annotation differs from this assignment

Supplementary figure 1. Simplified formation of SAM, L-asparagine and L-ornithine

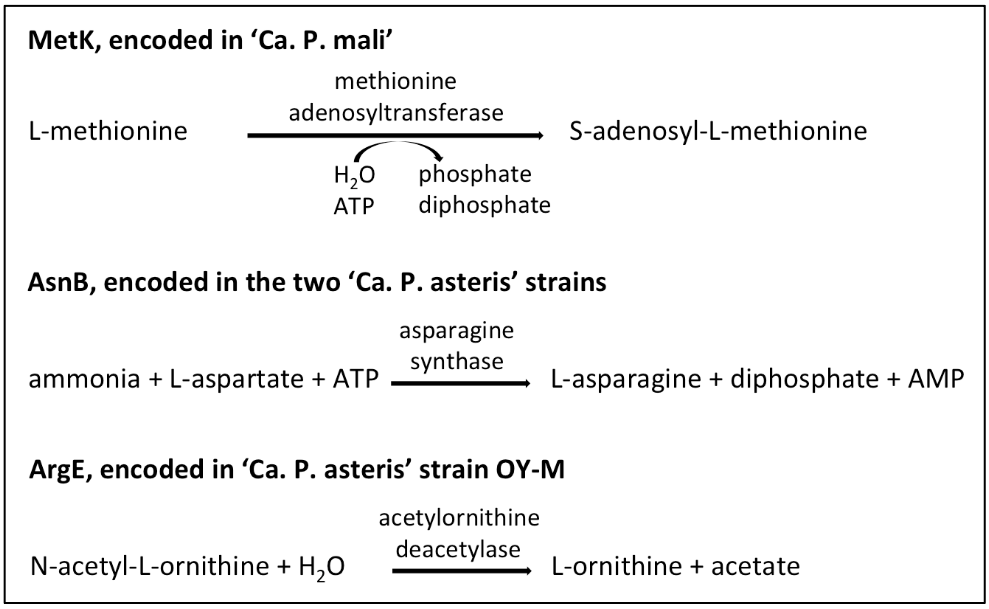

Supplement: Supplementary file 1 — Supplementary tables provide information on the gene content of “Ca. P. asteris” strains OY-M and AY-WB, “Ca. P. australiense” and “Ca. P. mali” according to functional categories and sequence homology. [file 185942.f1.pdf]
